# PHF20 stabilizes the GAS7-F-actin axis to drive DNA damage repair and chemoresistance in cutaneous squamous cell carcinoma

**DOI:** 10.1038/s41419-026-08932-6

**Published:** 2026-05-29

**Authors:** Yunqian Li, He Wen, Han Zheng, Yunyue Zhen, Jing Jia, Zhengjun Li

**Affiliations:** 1 Department of Dermatology, Qilu Hospital of Shandong University, Jinan, Shandong China; 2 Laboratory of Basic Medical Science, Qilu Hospital of Shandong University, Jinan, Shandong China; 3 Department of Orthopedics, The Second Qilu Hospital of Shandong University, Jinan, Shandong China; 4 Department of Pharmacy, Affiliated Hospital of Shandong University of Traditional Chinese Medicine, Jinan, Shandong China

**Keywords:** Oncogenes, Squamous cell carcinoma

## Abstract

Cutaneous squamous cell carcinoma (cSCC) is a common and aggressive skin cancer, with treatment options for advanced stages often limited by chemoresistance. Here, we observe a positive correlation between PHF20 expression and the malignancy and tumor grade of cSCC. Functional studies suggest that PHF20 promotes oncogenic phenotypes, including proliferation, invasion, and epithelial-mesenchymal transition. Notably, PHF20 depletion appears to sensitize cSCC cells to chemotherapeutic agents. Mechanistically, we show that PHF20 interacts with and promotes the ubiquitin-mediated degradation of GAS7. This downregulation of GAS7 is associated with nuclear F-actin assembly, a process that has been implicated in DNA damage repair (DDR). Consequently, PHF20 loss stabilizes GAS7, enhances nuclear F-actin assembly, and is accompanied by increased DNA damage accumulation. These in vitro findings were corroborated in vivo, where PHF20 knockdown suppressed tumor growth and increased DNA damage. Together, PHF20 may contribute to the regulation of DDR and chemotherapeutic response, highlighting its potential as a therapeutic target in poorly differentiated cSCC.

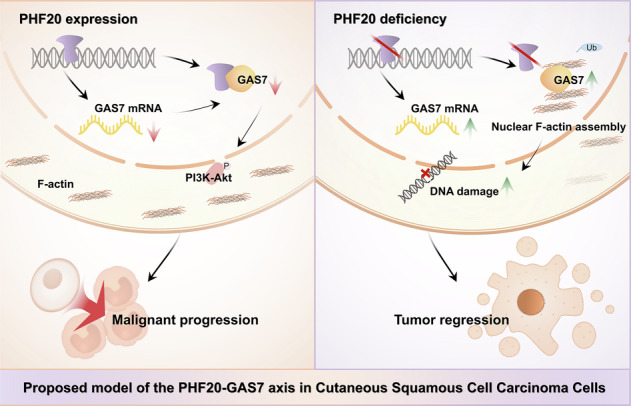

## Introduction

Nonmelanoma skin cancer is defined as one of the most common malignancies globally, with a higher incidence than melanoma [1, 2]. CSCC (cutaneous squamous cell carcinoma), making up 20–50% of nonmelanoma skin cancer cases, is characterized by rapidly increasing incidence, strong metastatic potential, and high recurrence rates [3, 4]. Commonly, patients with early-stage cSCC experience improved outcomes and benefit from advances in surgical resection and radiation therapy. However, therapeutic options for advanced and metastatic cSCC are limited, with chemoresistance representing a significant challenge clinically [5]. In general, cancer biology is primarily featured by the accumulation of DNA lesions, coupled with abnormal DDR (DNA damage repair) activity. Deficiency in DDR may induce genomic instability, resulting in increased mutations and tumor heterogeneity. Notably, oxidative stress represents a major endogenous source of DNA damage in skin-related disorders, and modulation of redox homeostasis has been shown to alleviate oxidative damage and improve disease outcomes [6]. All these characteristics may drive early tumorigenesis of cSCC [7, 8]. In the absence of correct repair, DSBs (DNA double-strand breaks) can trigger cell death or drive genomic instability, which makes them a particularly deleterious type of DNA lesion [9]. DSBs can be repaired through two main pathways, including the non-homologous end joining (NHEJ), which is error-prone and occurs in G1 phase, and homologous recombination (HR), which works in S and G2 phases to provide accurate repair using sister chromatids as a template [10]. Selection of the specific pathways depends on BRCA1, 53BP1, ATM, and other factors, which coordinate repair based on the cell cycle phase [11]. Conversely, once established, cSCC cells often exploit heightened DDR capacity to sustain proliferation and counteract therapy-induced DNA damage, ultimately fostering resistance to chemotherapy and radiotherapy [12, 13].

Repair proceeds within chromatin, where the kinetics and composition of repair assemblies at DSBs are dictated by the local accessibility and reader proteins. Recently, chromatin‑associated modulators of DDR have been identified by high‑content gain‑of‑function screening platforms that track factor recruitment to breaks. Notably, PHF20 has been suggested to influence the recruitment dynamics of DNA repair factors in chromatin contexts [14]. PHF20 has been implicated in transcriptional regulation, chromatin modification, and cell fate determination [15, 16]. Dysregulated PHF20 expression can promote tumorigenesis and correlate with poor prognosis, which has been reported in several malignancies, such as breast and colorectal cancer [17, 18].

Despite this emerging evidence, however, the role of PHF20 in cSCC remains poorly defined. Given the dualistic impact of DDR on tumor initiation versus treatment resistance, the present study proposed a hypothesis whereby PHF20 may contribute to pro‑survival DDR programs in advanced cSCC. Mechanistically, we further considered GAS7 as a potential downstream effector. GAS7 is a key regulator of the cytoskeleton, which is capable of binding to F-actin and microtubules to promote cell membrane protrusions and changes in cell morphology [19–21]. The C-terminal domain of GAS7 mediates actin polymerization, which can interact with the N-WASP/Arp2/3 complex to modulate microfilament rearrangement [21, 22]. Downregulation or loss of GAS7 has been documented to enhance tumor cell migration and invasion in various cancers, whereas its expression can be directly regulated by p53 [22, 23]. In this study, we aimed to explore the role and mechanism of PHF20 in poorly differentiated cSCC through enhanced DDR.

## Materials

### Patient samples

Human samples were collected with informed consent and ethical approval from Sino Bright (Xi’an) Intelligent Biotechnology Co., Ltd. and Qilu Hospital of Shandong University (Approval no. KYLL-2021(KS)-90). A tissue microarray (K054Sk01) comprising 38 cSCC, 9 peritumoral, and 7 normal skin tissues was obtained from Sino Bright (Xi’an). Fresh surgical samples were fixed in 4% PFA and embedded in paraffin. Tumor grading followed the WHO SCC criteria: Grade 1 (75–100% differentiation), Grade 2 (50–75%), and Grade 3 (0–50%) [24].

### Cell lines and primary keratinocyte culture

The human cSCC cell lines A431 (ATCC CRL-1555; RRID: CVCL_0037) and SCL-1 (RRID: CVCL_A789) were cultured in Dulbecco’s modified Eagle’s medium (DMEM; Gibco), supplemented with 10% fetal bovine serum (FBS; Gibco) and 1% penicillin–streptomycin (Gibco) at 37°C in a humidified atmosphere containing 5% CO₂. A431 cells were purchased from ATCC, and SCL-1 cells were generously provided by the Xiangya School of Medicine (Changsha, China) in 2023. Both A431 and SCL-1 cell lines are *TP53*-deficient due to reported inactivating alterations [25, 26].

Primary normal human epidermal keratinocytes (NHEKs) were isolated from neonatal foreskin samples after providing informed consent. Briefly, the epidermis and dermis were separated by dispase II treatment (4°C, overnight), followed by 0.25% trypsin-EDTA digestion to obtain single-cell suspensions. NHEKs were cultured in keratinocyte-specific medium (Xirui Wuxian Biotechnology Co., Ltd., Hangzhou, China), and cells at passage five were used for all experiments. All the cell lines were tested negative for mycoplasma contamination via a PCR-based assay before experimental use.

### Cell transfection

A431 and SCL-1 cells were transfected with siRNAs (RiboBio, Guangzhou, China) and GAS7 cloned into pcDNA3.1-MCS-P2A-mCherry-Flag-Puro (Bioosen, Shanghai, China) via the NB Transfection Reagent (Bioosen, Shanghai, China) according to the manufacturer’s instructions. The siRNAs target sequences are listed in Table 1.

**Table 1:** List of siRNA target sequences used for gene knockdown in cSCC cells.

siRNA	Species	Target sequence (5’-3’)
si-PHF20-1	Human	GGAGAAAACACGAUGAAAAtt
si-PHF20-2	Human	CUACAAAAGACAAGGAAAAtt
si-GAS7-1	Human	GGAGCUACUGCGACUACUUtt
si-GAS7-2	Human	CCCAGUCCAAAUGGUUUGAtt
si-Control	Human	UUCUCCGAACGUCUCACGUtt

### HR/NHEJ reporter assays

Homologous recombination (HR) and non-homologous end joining (NHEJ) repair efficiencies were assessed using the DR-GFP and EJ5-GFP reporter systems, respectively [27, 28]. A431 and SCL-1 cells were first transfected with control or PHF20-targeting siRNAs. Twenty-four hours later, cells were co-transfected with pDR-GFP (#17617, Addgene) or pimEJ5-GFP (#44026, Addgene) with the I-SceI expression plasmid pCBASceI (#26477, Addgene) to induce site-specific double-strand breaks. After 8 h of transfection, cells were incubated with etoposide and then harvested 48 h after transfection to be analyzed by flow cytometry. All experiments were performed in biological triplicate, and data are presented as mean ± SD.

### 3D tumor spheroid formation assay

A431 and SCL-1 cells (5000 cells/well) were seeded in ultralow attachment 96-well U-bottom plates (Corning Costar, USA) in 100 μL of serum-free DMEM. Spheroid formation was monitored daily, and the samples were imaged via a bright-field microscope (Olympus, Japan). On day 3, mature spheroids were established and treated with cisplatin (10 μM, Sigma-Aldrich, St. Louis, USA) or etoposide (20 μM, Sigma-Aldrich, St. Louis, USA) for an additional 48 hours. The experiments were performed in triplicate and repeated independently three times.

### DNA fiber assay

DNA fiber analysis was performed to assess replication fork dynamics as previously described [29, 30]. Cells were pulse-labeled with 50 μM 5-iodo-2′-deoxyuridine (IdU, HY-112669, MCE) for 20 min to label ongoing replication forks, followed by treatment with etoposide for 60 min to induce DNA damage. Subsequently, cells were labeled with 100 μM 5-chloro-2′-deoxyuridine (CIdU, HY-B0307, MCE) for an additional 20 min to monitor replication restart or fork progression after damage. After labeling, approximately 200 cells were spotted onto glass slides. Cells were lysed with spreading buffer (200 mM Tris-HCl, pH 7.4, 50 mM EDTA, 0.5% SDS) for 10 min. DNA fibers were then stretched by tilting the slides at a 25° angle and air-dried for 6 h. Slides were fixed in methanol/acetic acid (3:1), and dried overnight with limited light exposure, followed by storage at −20 °C for 48 h.

Prior to immunostaining, slides were briefly defrosted and immersed in deionized water for 20 s, then DNA fibers were denatured with 2.5 M HCl for 80 min at room temperature. After extensive washing with PBS, slides were blocked with 5% BSA and incubated with primary antibodies against CldU (rabbit anti-BrdU, clone YA578, HY-P80038, MCE) and IdU (mouse anti-IdU, D199955-0100, Sangon Biotech) for 2 h at room temperature. Following washing, appropriate fluorophore-conjugated secondary antibodies were applied. DNA fibers were visualized using an oil-immersion objective on a fluorescence microscope.

### Coimmunoprecipitation (co-IP)

Cells were lysed and incubated with Protein A/G PLUS-Agarose beads (MCE, Monmouth Junction, NJ, USA) on ice for 30 minutes for preclearing. Subsequently, anti-PHF20 and anti-GAS7 antibodies were added and incubated overnight at 4 °C with rotation. Fresh beads captured the immunocomplexes, which were then washed extensively and subjected to Western blot analysis. Rabbit or mouse IgG served as a negative control.

### Ubiquitination assay

A431 and SCL-1 cells were transfected with control siRNA or PHF20-specific siRNA. 48 hours post-transfection, the cells were treated with MG132 (10 μM, Sigma-Aldrich, St. Louis, USA) for 6 hours to inhibit proteasome-mediated degradation. The cells were lysed in an NP-40 protease inhibitor cocktail, and the lysates were precleared and immunoprecipitated with anti-PHF20 and anti-GAS7 antibodies overnight at 4 °C. Protein G agarose beads (MCE, Monmouth Junction, NJ, USA) were added to the mixture, and it was incubated for 2 hours. After extensive washing, the immunoprecipitates were subjected to SDS‒PAGE and immunoblotted with anti-ubiquitin and anti-GAS7 antibodies to detect ubiquitinated GAS7. The input levels of GAS7 and PHF20 were confirmed by Western blot analysis. Parallel experiments using cycloheximide (CHX, 100 μg/mL, Sigma-Aldrich, St. Louis, USA) chase were performed to assess the stability of GAS7.

### In Vivo Xenograft model

Male BALB/c nude mice (4–5 weeks old) were subcutaneously injected with 5 × 10⁶ shRNA-transfected A431 or SCL-1 cells suspended in PBS. Mice were randomly assigned to treatment and control groups using a computer-generated randomization schedule (*n* = 5). Tumor volumes were measured every 3 days by an investigator blinded to group allocation, and tumor volume was calculated via the following formula: volume = (length × width²)/2. At the endpoint, the tumors were excised for protein analysis and subsequently embedded in paraffin. All animal experiments were conducted under the approval of the Institutional Animal Care and Use Committee of Qilu Hospital of Shandong University (Approval: DWLL-2024-314), following NIH guidelines. No animals were excluded from the analysis, and no unexpected adverse events were observed. Sample sizes were based on previous studies and feasibility, while minimizing animal use in accordance with ethical guidelines; no formal power analysis was conducted.

### Statistical analysis

Sample sizes were determined based on prior experience with similar experimental systems and published studies in the field, and were sufficient to detect biologically meaningful differences between groups. No statistical methods were used to predetermine sample size. All experiments were independently repeated at least three times, and the data are expressed as the means ± standard deviation (S.D.), unless otherwise specified. An independent unpaired two-tailed Student’s *t*-test was performed to evaluate the differences between the two groups. Multiple group comparisons were analyzed using one-way analysis of variance (ANOVA) with Bonferroni correction. *P* < 0.05 was considered statistically significant. All the analyses were conducted via GraphPad Prism 9.

Other methods not described here are available in the Supplementary material.

## Results

### High PHF20 expression was correlated with the malignancy and tumor grade of cSCC

**Fig. 1:**
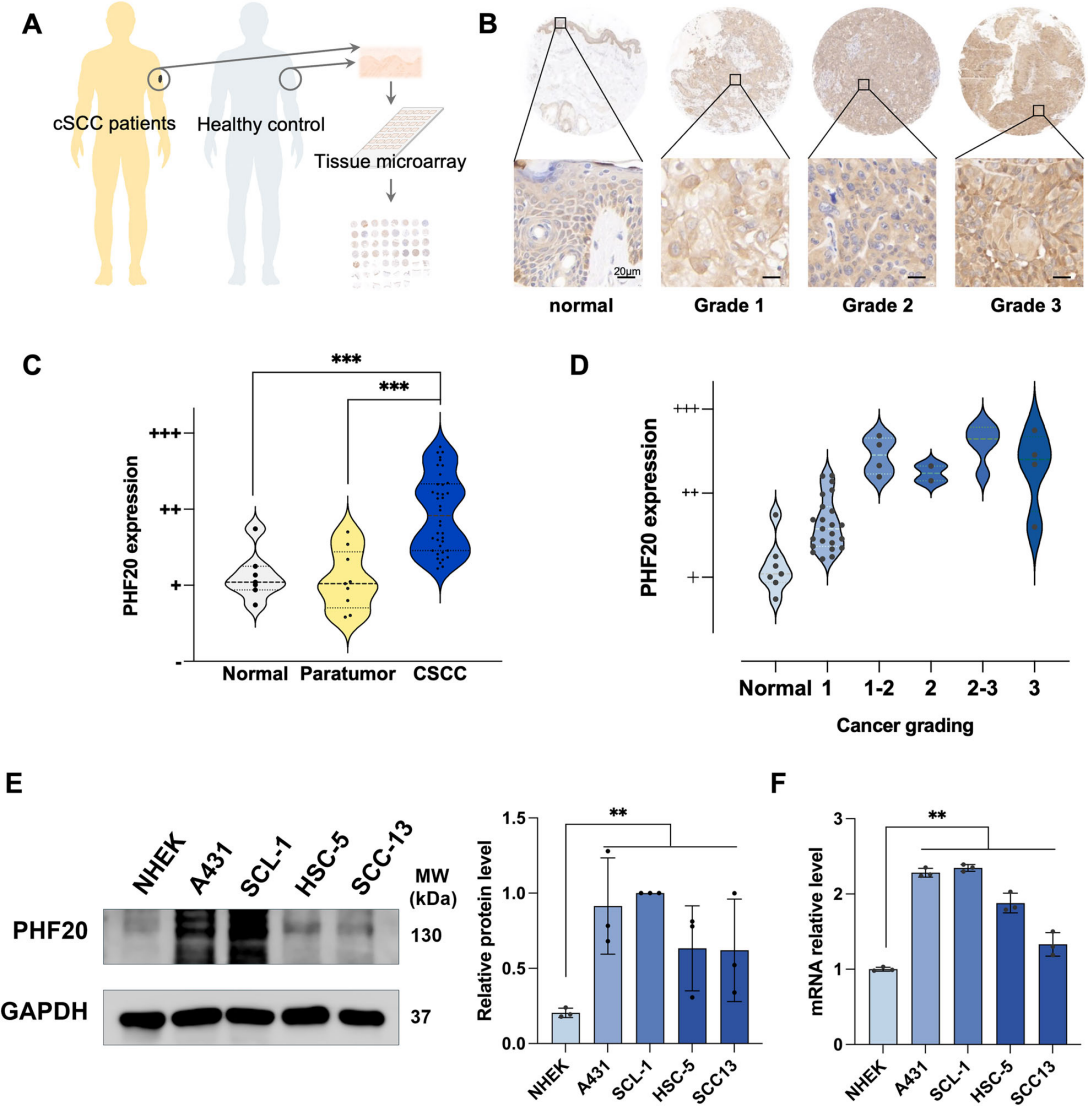
High PHF20 expression was correlated with the malignancy and tumor grade of cSCC. **A** Overview of the tissue microarray study design. **B** Representative images of PHF20 staining by immunohistochemistry in normal skin and different grades of cSCC. Bar = 20 μm. **C** Quantification of the IHC score of PHF20 in normal tissues, paratumor tissues and cSCC tumor tissues. **D** Association of PHF20 staining score with tumor grade (normal skin tissues, I, II, III & IV). **E** Western blot and quantification of PHF20 levels in NHEK and cSCC cell lines. **F** RT‒qPCR analysis of PHF20 levels in NHEK and cSCC cell lines. The data are shown as the means ± SDs from three independent experiments. **p < 0.01, and ***p < 0.001.

To investigate the role of PHF20 in cSCC, IHC (immunohistochemistry) was conducted initially to assess PHF20 immunoreactivity in cSCC samples (*n* = 38), perilesional tissues (*n* = 9), and normal skin tissues (*n* = 7) (Fig. 1A, Table 2). The majority of cSCC samples presented a strong PHF20 signal (Fig. 1B, C). Notably, PHF20 staining was detected in both the nucleus and cytoplasm. Such heterogeneous subcellular distribution has also been observed for PHF20 in different tumor types [31–33]. There, high-grade tumors frequently showed enhanced nuclear–cytoplasmic co-localization of PHF20. Based on further analysis of the IHC-stained sections, there was a positive correlation between PHF20 immunoreactivity and increasing cSCC grade (Fig. 1D, Table 3). Additionally, detection at both the mRNA and protein levels indicated that the expression of PHF20 was elevated in cSCC cell lines compared with normal human epidermal keratinocytes (Fig. 1E, F). Collectively, these results indicate that elevated PHF20 expression is closely associated with cSCC malignancy and poor differentiation, suggesting a potential oncogenic role.

**Table 2:** Baseline characteristics of study participants.

	Healthy (*n* = 7)	Paratumor (*n* = 9)	CSCC (*n* = 38)
**Age, y, mean (SD)**	41.9 (6.98)	61.6 (12.7)	61.2 (13.6)
**Male** **sex**, *n* **(%)**	71.43	22.22	52.63
**Tumor Stage,** *n* **(%)**			
Ⅰ			13.16
Ⅱ			60.53
Ⅲ			15.79
Ⅳ			5.26
TMN, *n* (%)			
T1M0N0			13.16
T2M0N0			55.26
T2M1-2N0			5.26
T3M0N0			15.79
T4M0Nx			2.63
Cancer grading, *n* (%)			
1			60.53
1~2			10.53
2			5.26
2~3			18.42
3			10.53

**Table 3:** PHF20 staining of all CSCCs (*n* = 38).

Variables	Gray level value mean (SD)	Total	*P* Value
**Age, y**			
>=60	299,391.25 (85,830.42)	28	0.4186
<60	278,576.02 (67,667.48)	26	
**Sex**			
Male	281,284.98 (71,834.82)	28	0.3496
Female	303,319.71 (75,284.41)	26	
Tumor Stage, *n* (%)			
Ⅰ	297,421.21 (57,856.84)	5	0.0584
Ⅱ	267,746.63 (65,905.86)	22	
Ⅲ	333,210.40 (71,895.18)	7	
Ⅳ	377,283.66 (45,801.07)	2	
TMN, *n* (%)			
T1	297,421.21 (57,856.84)	5	0.1155
T2	274,601.50 (67,283.64)	23	
T3	327,324.23 (76,078.08)	6	
T4	423,084.73 (0)	1	
Cancer grading, *n* (%)			
1	244,439.23 (45,602.54)	23	<0.0001
1-2, 2	356,435.05 (28,971.06)	6	
2-3, 3	369,415.74 (53,834.79	9	

### PHF20 maintained the tumor-promoting role of cSCC

**Fig. 2:**
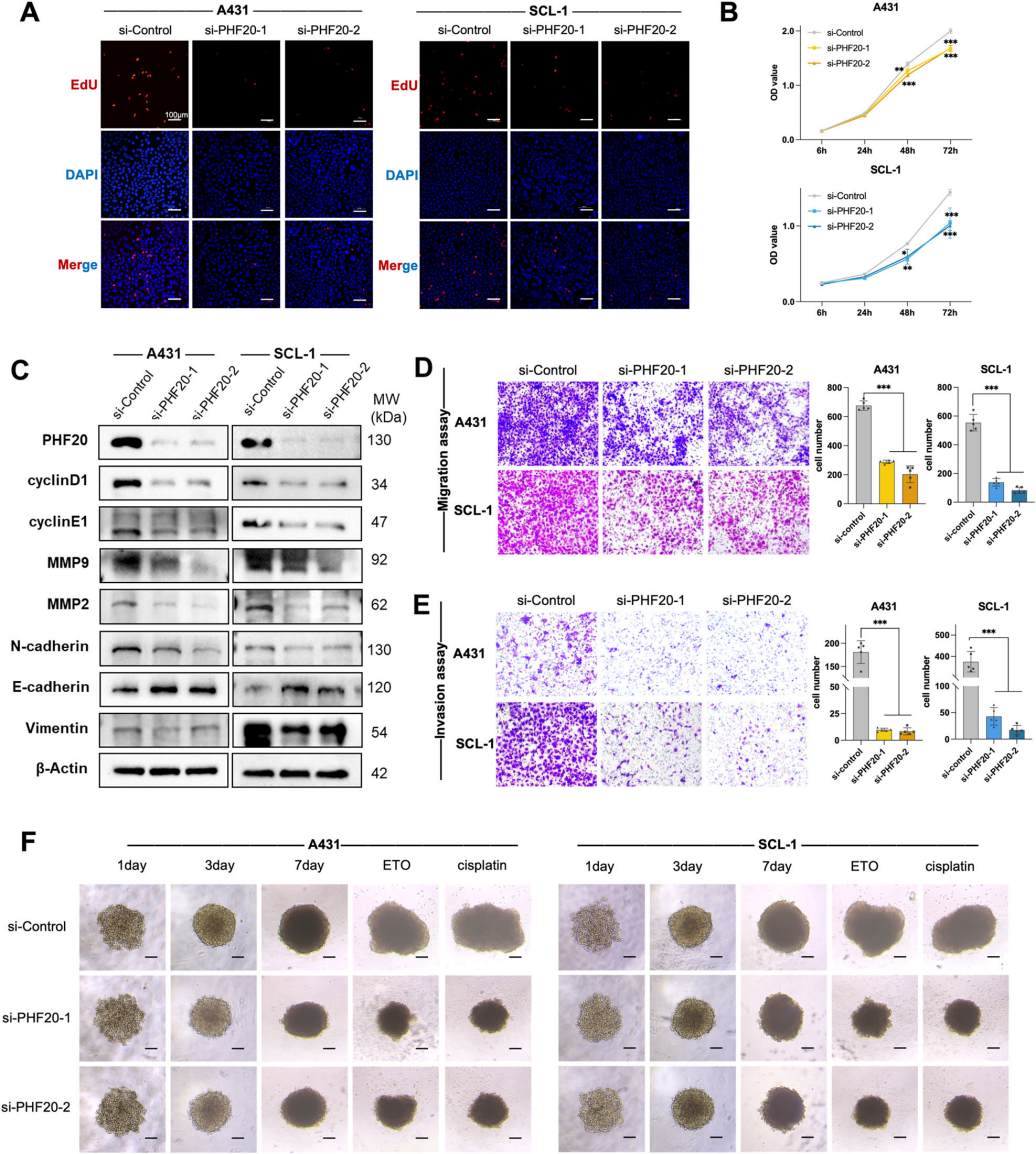
PHF20 maintained the tumor-promoting role of cSCC. **A** Representative fluorescence images of the EdU (red) assay in PHF20-depleted cSCC cells. Nuclei were stained with DAPI (blue), and a combined reaction involving EdU and DAPI indicated the number of proliferating cells. Bar=100 μm. **B** CCK-8 assays of PHF20-depleted cSCC cells. **C** Western blot analysis of proliferation-, migration-, invasion- and EMT-related marker expression in PHF20-depleted cSCC cells. **D** Transwell assay and quantification of PHF20 depletion on cSCC cell migration ability. **E** Transwell assay with Matrigel and quantification of the invasive ability of PHF20-depleted cSCC cells. **F** Sensitivity assays of PHF20-depleted cells treated with the indicated doses of ETO and cisplatin for 48 hours. Upon treatment with ETO or cisplatin for 48 hours, spheroids derived from PHF20-depleted cells exhibited pronounced shrinkage and apoptosis. Bar = 20 μm. The data are shown as the means ± SDs from three independent experiments. *p < 0.05, **p < 0.01, and ***p < 0.001.

Using small interfering RNA (siRNA), we constructed PHF20-depleted cSCC cells and assessed their efficiency in investigating its role in the malignant phenotype (Fig. S1A, B). Knocking down PHF20 expression significantly impaired cell proliferation (Fig. 2A, B, S1C), and reduced protein levels of Cyclin D1 and Cyclin E1 (Fig. 2C). Furthermore, wound-healing and Transwell assays revealed that PHF20 depletion substantially attenuated the migration and invasive capacities of cSCC cells (Fig. 2D, E, S1D, E). Consistent with these findings, the expression of metastasis-associated proteins MMP-2 and MMP-9 was remarkably decreased (Fig. 2C). We then examined key epithelial-mesenchymal transition (EMT) markers, given that EMT endows epithelial cells with migratory and invasive properties [34]. PHF20 depletion triggered an increase in E-cadherin expression, while simultaneously decreasing the expression of N-cadherin and vimentin (Fig. 2C). Notably, beyond suppressing basal oncogenic phenotypes, PHF20 depletion profoundly sensitized cSCC cells to genotoxic chemotherapeutic agents, including etoposide (ETO) and cisplatin (Fig. 2F, S1F). This prompted us to investigate whether PHF20 modulates DDR, a key determinant of chemoresistance.

### Loss of PHF20 impairs DDR and induces apoptosis

**Fig. 3:**
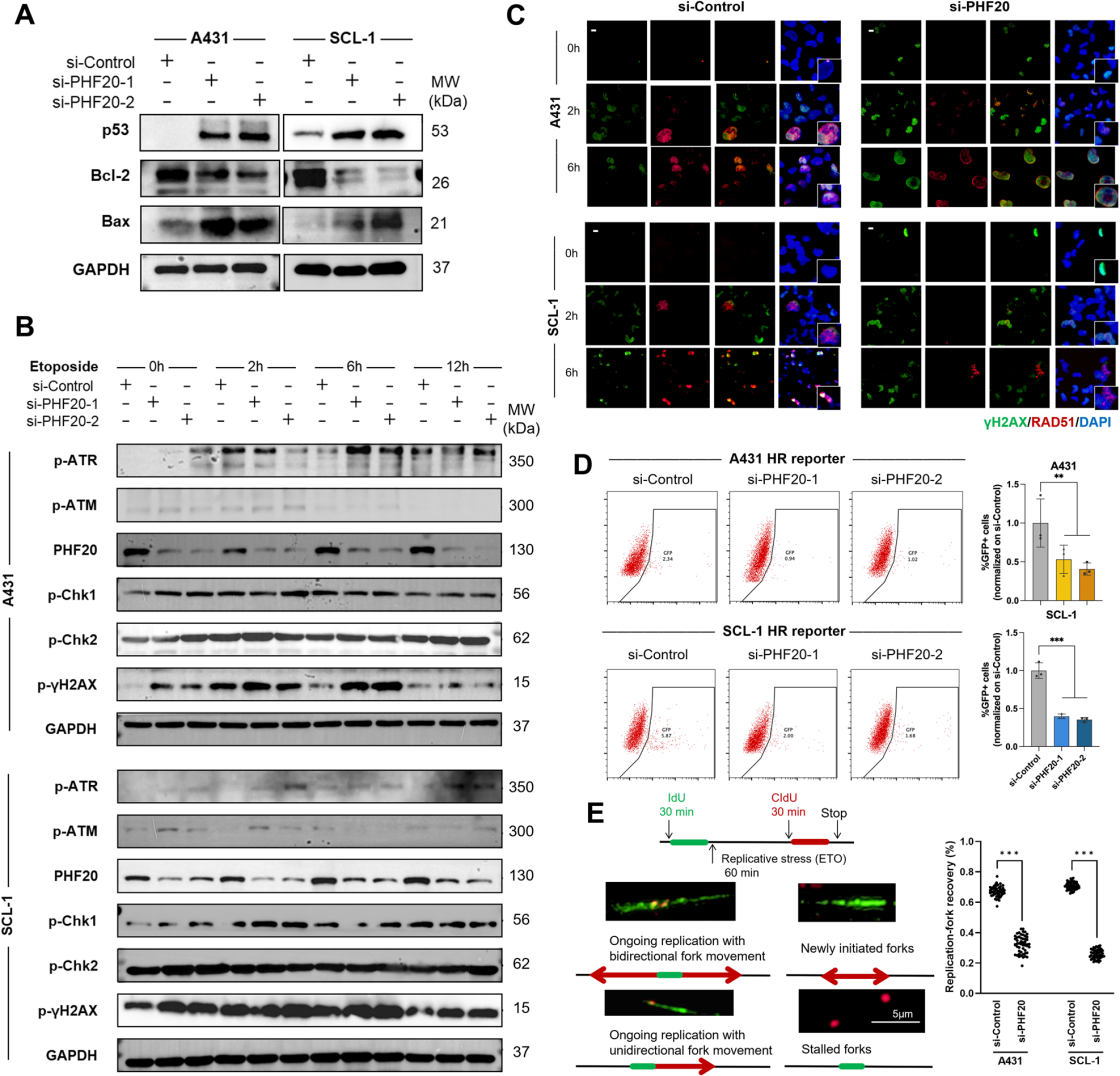
Loss of PHF20 impairs DDR and induces apoptosis. **A** Western blot analysis of p53, Bcl-2 and Bax after PHF20 depletion. **B** PHF20-depleted A431 or SCL-1 cells were treated with ETO. The levels of p-ATM, p-ATR, p-Chk1/2, and γH2AX were analyzed by Western blotting. **C** Representative immunofluorescence analysis of γH2AX and RAD51 foci formation following DNA damage. Bar = 10 μm. **D** Quantification of HR repair efficiency using DR-GFP reporter assays **E** DNA fiber assay assessing replication fork progression and restart following etoposide treatment. The data are shown as the means ± SDs from three independent experiments. **p **<** 0.01, and ***p **<** 0.001.

Given that PHF20 loss induced chemosensitivity and marked S-phase accumulation (Fig. S2A), a cellular phenotype commonly associated with elevated replication stress [35, 36], we further examined its involvement in DDR. Micronuclei formation has been adopted as a common method to monitor genotoxic events and chromosomal instability [37]. PHF20 depletion in cSCC cells increased the proportion of micronuclei, in the context of with or without bleomycin treatment (Fig. S2B). Consistent with this, PHF20 depletion led to a significant increase in both early and late apoptotic cell populations (Fig. S2C). At the molecular level, this was associated with upregulated expression of pro-apoptotic genes and proteins, alongside downregulation of anti-apoptotic factors (Figs. 3A, S3D).

Upon etoposide exposure, PHF20-deficient cells exhibited a markedly delayed resolution of the γH2AX signal, suggesting persistent DNA damage. To further characterize how PHF20 loss affects the DNA damage response, we examined the phosphorylation of ATM, ATR, CHK1, and CHK2. Time-course revealed altered activation dynamics of core DDR checkpoint pathways, indicating that PHF20 depletion perturbs DDR signaling cascades (Fig. 3B). Notably, a modest increase in basal ATR–CHK1 phosphorylation was observed in PHF20-depleted cells at 0 h, which is consistent with the increased S-phase accumulation detected under the same conditions.

Since DDR signaling ultimately governs the recruitment of pathway-specific repair factors [38], we next assessed the assembly of key DSB repair mediators by immunofluorescence. PHF20-deficient cells displayed a pronounced and prolonged accumulation of γH2AX following DNA damage. This was accompanied by impaired recruitment of DNA repair factors, characterized by significantly reduced RAD51 foci formation at later time points, together with altered 53BP1 foci dynamics (Figs. 3C, S2D). To gain mechanistic insights into this DDR deficiency, we performed transcriptomic analysis (Fig S3A–C). Gene set enrichment analysis (GSEA) corroborated these findings, showing a negative enrichment for DSB repair pathways and a positive enrichment for DNA repair defect pathways (Fig. S3E) upon PHF20 knockdown. The accumulation of unresolved DNA damage ultimately triggers cell fate decisions. To directly evaluate whether PHF20 affects the efficiency of major DSB repair pathways, we employed DR-GFP/EJ5-GFP reporter assays. PHF20 knockdown resulted in reduction in both HR and NHEJ efficiencies, indicating an overall impairment of DSB repair capacity (Figs. 3D, S2E). Consistent with compromised DSB repair, DNA fiber assays further revealed impaired replication fork progression and restart following etoposide treatment in PHF20-deficient cells (Fig. 3E), indicating compromised fork stability under genotoxic stress [39, 40]. Together, these findings support a model in which PHF20 loss is associated with impaired DNA repair and increased replication stress.

### PHF20 interacts with and destabilizes GAS7 via the ubiquitin-proteasome pathway

**Fig. 4:**
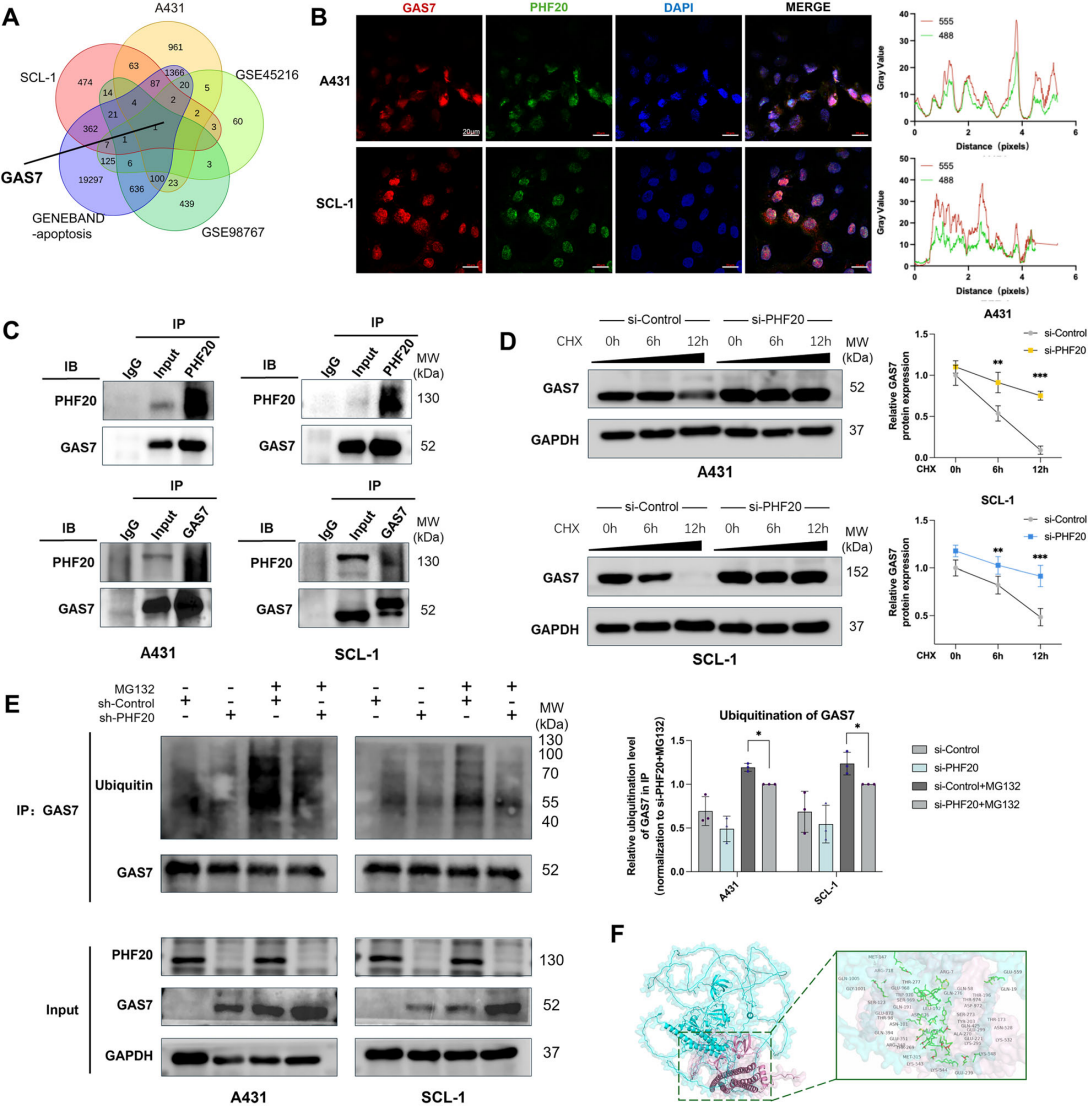
PHF20 interacts with and destabilizes GAS7 via the ubiquitin-proteasome pathway. **A** Venn diagram showing the intersection between RNA-seq data and public databases. **B** Representative multiplex immunofluorescence (mIHC) staining of PHF20 (green) and GAS7 (red) in cSCC cells. Nuclei were counterstained with DAPI (blue). Bar = 20 μm. **C** Co-IP assays showing the interaction between PHF20 and GAS7. Immunoprecipitation was performed using anti-PHF20 and anti-GAS7 antibody, with normal rabbit IgG and mouse IgG used as a negative control. **D** A cycloheximide (CHX) chase assay demonstrated enhanced GAS7 protein stability following PHF20 knockdown. **E** Ubiquitination of GAS7 analyzed by immunoprecipitation followed by immunoblotting with anti-ubiquitin antibody in control and PHF20-depleted cells. **F** Structural characterization of the PHF20-GAS7 interaction interface. Key residues form hydrogen bonds (yellow dashed lines) and salt bridges. Distance measurements performed in PyMOL (Å). The data are shown as the means ± SDs from three independent experiments. *p **<** 0.05, **p **<** 0.01, and ***p **<** 0.001.

Our transcriptomic analysis of cSCC cell lines and public datasets (GSE45216 and GSE98767) consistently showed that PHF20 knockdown led to upregulation of GAS7, which was conversely downregulated in cSCC patient samples (Figs. 4AS4A) [41]. These findings were further confirmed by quantitative polymerase chain reaction and Western blot (Fig. S4B, C). Given prior evidence that PHF20 modulates DNA damage responses predominantly at the protein level, we hypothesized that PHF20 may regulate GAS7 through a post-translational mechanism. Subcellular localization analysis revealed the predominant nuclear distribution of both PHF20 and GAS7, as supported by nuclear/cytoplasmic fractionation and immunofluorescence (Figs. 4B, S4D). And a direct physical interaction between PHF20 and GAS7 was confirmed by co-immunoprecipitation assays (Fig. 4C).

In general, protein-protein interaction-mediated degradation typically involves either lysosomal (autophagic) or proteasomal (ubiquitin-dependent) pathways [42]. Annotation in the PhosphoSitePlus database indicated ubiquitin-mediated regulation of GAS7 (Fig S4E). CHX-chase assays showed that PHF20 depletion prolonged the half-life of GAS7 protein (Figs. 4D, S4F). Ubiquitination assays showed that PHF20 depletion was associated with reduced ubiquitination of GAS7, consistent with a role for PHF20 in promoting GAS7 ubiquitination and proteasome-dependent degradation (Fig. 4E). To define the interaction interface, we performed molecular docking analysis. The most stable hydrogen bond interaction was observed between Gln276 of PHF20 and Gln58 of GAS7 (ΔG = –9.8 kcal/mol; distance: 2.10 Å) (Fig. 4F, Table 4), located near the SH3 domain of GAS7, which is an evolutionarily conserved motif known for mediating protein–protein interactions [43]. This interaction was further supported by “SH3 domain binding” in PHF20-depleted cells GO enrichment analysis (Fig. S3B). Additionally, several strong salt bridges were observed (Table 5). These electrostatic interactions occurred in proximity to predicted ubiquitination sites on GAS7 (K257, K279, and K360). Hence, PHF20 binding may influence the structural conformation or accessibility of these lysine residues, potentially contributing to ubiquitin-mediated degradation.

**Table 4:** Hydrogen bonds interactions between PHF20 and GAS7, distances (Å) were measured using PyMOL.

PHF20	GAS7	Dist. [Å]
ALA 270 [N]	GLU 299 [OE1]	3.34
SER 273 [OG]	THR 98 [O]	3.7
SER 273 [OG]	ASN 101 [OD1]	3.82
THR 277 [N]	GLN 58 [OE1]	3.51
ASN 528 [ND2]	GLU 351 [OE1]	3.8
LYS 532 [NZ]	ARG 340 [O]	2.93
LYS 543 [NZ]	MET 315 [O]	2.73
LYS 544 [N]	GLU 221 [OE1]	3.84
LYS 548 [NZ]	GLU 239 [OE2]	3.37
ARG 718 [NH1]	MET 147 [O]	3.55
ARG 718 [NH2]	MET 147 [O]	3.46
SER 969 [OG]	GLN 191 [OE1]	3.34
TRP 970 [N]	LEU 192 [O]	3.78
**THR 974 [N]**	**TYR 203 [OH]**	**2.17**
**GLN 1005 [NE2]**	**SER 123 [OG]**	**2.25**
THR 269 [O]	GLN 425 [NE2]	3.72
ALA 270 [O]	LYS 295 [NZ]	3.53
VAL 271 [O]	LYS 295 [NZ]	3.72
**GLN 276 [O]**	**GLN 58 [NE2]**	**2.1**
GLN 276 [OE1]	ARG 7 [NH1]	2.9
GLU 559 [OE2]	GLN 19 [NE2]	3.46
GLU 873 [OE1]	GLN 394 [NE2]	3.79
ASP 876 [OD2]	THR 173 [OG1]	3.89
ASP 876 [OD2]	THR 173 [N]	3.76
**GLU 968 [OE1]**	**THR 196 [OG1]**	**2.69**
SER 969 [O]	LEU 192 [N]	2.94
ASP 972 [OD1]	LEU 192 [N]	3.88
GLY 1001 [O]	SER 123 [OG]	3.54

**Table 5:** Salt bridges interactions between PHF20 and GAS7, distances (Å) were measured using PyMOL.

PHF20	GAS7	Dist. [Å]
LYS 543 [NZ]	ASP 319 [OD2]	2.96
LYS 548 [NZ]	GLU 239 [OE2]	3.37
LYS 738 [NZ]	GLU 189 [OE2]	2.81

### GAS7 downregulation is required for PHF20-mediated DDR and chemoresistance

**Fig. 5:**
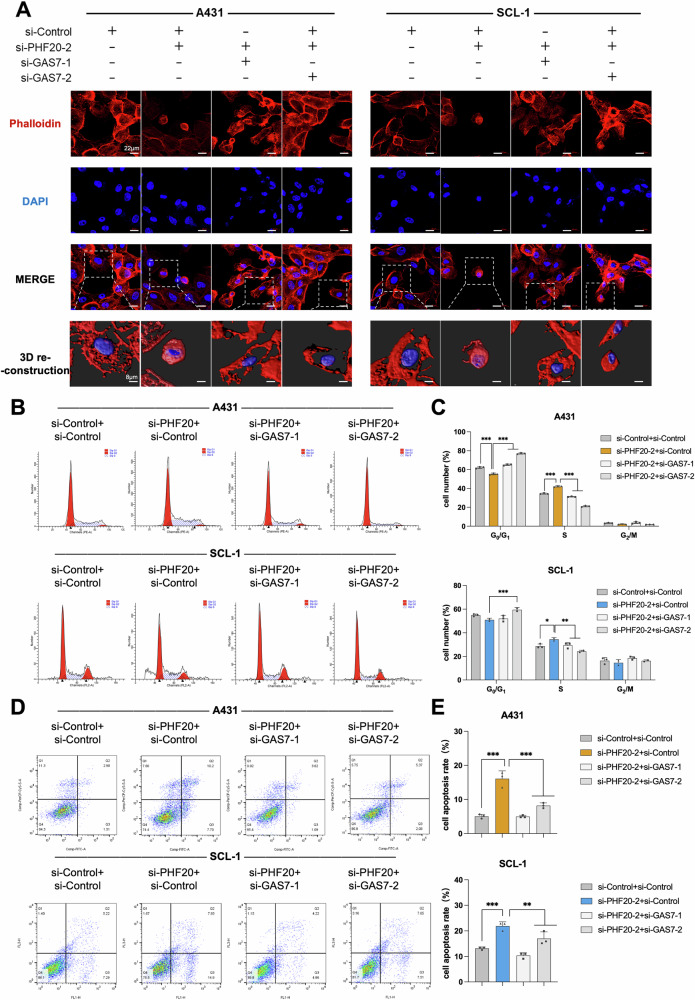
GAS7 downregulation is required for PHF20-mediated DDR and chemoresistance. **A** Representative confocal microscopy images of F-actin (phalloidin, red) in cSCC cells transfected with control siRNA, PHF20 siRNA, or co-transfected with PHF20 and GAS7 siRNAs. Nuclei were counterstained with DAPI (blue). 3D reconstruction of nuclear F-actin was performed based on Z-stack images using IMARIS software. **B**, **C** Flow cytometry analysis of the cell cycle and quantification at 48 hours after PHF20 depletion with or without GAS7 depletion. **D**, **E** Flow cytometry analysis of apoptosis and quantification at 48 hours after PHF20 depletion with or without GAS7 depletion. The data are shown as the means ± SDs from three independent experiments. **p < 0.01, and ***p < 0.001.

To investigate whether this regulation was required for the antiapoptotic role of PHF20, PHF20 and GAS7 were simultaneously knocked down via siRNAs (Fig. S5A, B). In the nucleus, nuclear F-actin remains a major factor in DNA repair kinetics, including chromatin movement, DSB relocalization, and DDR efficiency [21, 44, 45]. GAS7, particularly its full-length and C-terminal domains, can directly bind and promote the polymerization of F-actin [19, 46, 47]. Following PHF20 knockdown, there was a marked increase in the staining intensity of nuclear F-actin compared with control group, whereas co-transfection with siGAS7 reversed this phenotype (Fig. 5A). Enhanced F-actin polymerization in the nuclei of PHF20-deficient cells was further confirmed by three-dimensional reconstruction of Z-stack images using IMARIS. Furthermore, Quantitative analysis of phalloidin fluorescence confined to LaminB1-defined nuclear regions confirmed a robust increase in nuclear F-actin upon PHF20 depletion, which was markedly attenuated by GAS7 knockdown (Fig. S6A). Consistently, nuclear and cytoplasmic fractionation followed by immunoblotting was performed. PHF20 silencing resulted in a pronounced increase of β-actin in the nuclear fraction, while cytoplasmic β-actin levels were not significantly altered. Notably, concomitant GAS7 knockdown reduced nuclear β-actin accumulation, supporting a GAS7-dependent regulation of nuclear actin dynamics downstream of PHF20 (Fig. S6B).

These data suggest that PHF20 suppresses nuclear F-actin polymerization by downregulating GAS7. Consequently, we hypothesized that GAS7 downregulation is required for PHF20’s effects on DDR. Indeed, GAS7 knockdown partially reversed the PHF20 depletion-induced S cell cycle arrest (Fig. 5B, C), partially reversed apoptosis (Fig. 5D, E), and alleviated the number of DNA end breaks (Fig. S5C, D). Based on western blot analysis, GAS7 knockdown restored p53 expression and reactivated the PI3K/Akt pathway, which was highlighted by Kyoto Encyclopedia of Genes and Genomes enrichment analysis (Figs. S5B, S3C). Together, these rescue experiments demonstrate that the downregulation of GAS7 is functionally required for PHF20-mediated DDR.

### Loss of PHF20 suppressed tumor growth and promoted DNA damage-mediated apoptosis in vivo

**Fig. 6:**
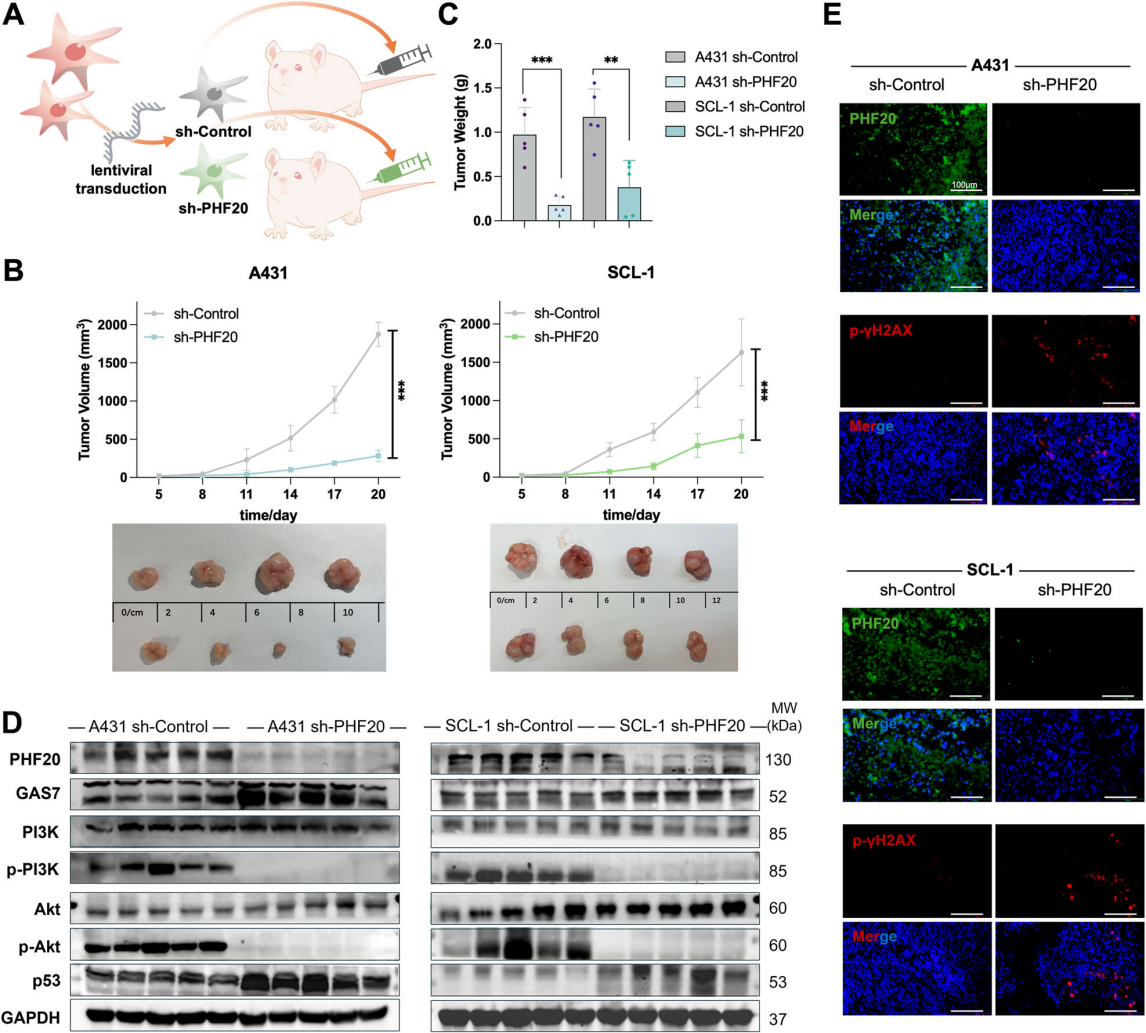
Loss of PHF20 suppressed tumor growth and promoted DNA damage-mediated apoptosis in vivo. **A** Schematic illustration showing whether PHF20 enhances cSCC promotion in vivo. **B** The weights of the tumors harvested at the end of the experiment (*n* = 5). **C** Pictures of the tumors harvested and tumor growth of nude mice. **D** Western blot analysis of PHF20, PI3K/Akt signaling, and apoptosis markers in xenografts (*n* = 3). **E** Representative images of immunofluorescence staining for PHF20 (green) and p-γ-H2AX (red) in xenograft tumor samples. Nuclei were counterstained with DAPI (blue). Bar=100 μm. The data are shown as the means ± SDs from at least four independent experiments. **p < 0.01, and ***p < 0.001.

To explore the in vivo relevance of PHF20 in cSCC progression, immunocompromised mice were used for establishing a xenograft tumor model (Fig. 6A). From day 11 onward, PHF20 depletion led to a marked decrease in xenograft size (Fig. 6B) and a statistically significant reduction in tumor burden at the end of the evaluation (Figs. 6C, S7A). Western blot and immunofluorescence confirmed the efficiency of PHF20 knockdown in the xenografts, which was accompanied by increased GAS7 expression, supporting their negative regulatory relationship (Fig. 6D, E). Importantly, with PHF20 depleted, it was observed with a significant reduction in the phosphorylation of the PI3K/Akt axis and the antiapoptotic protein Bcl-2; the upregulation of GAS7, p53, the proapoptotic protein Bax; and the cleavage of caspases (i.e., Caspase-7 and Caspase-3) (Figs. 6DS7B). The fluorescence results revealed elevated γ-H2AX and GAS7 in tumor tissues (Figs. 6E, S7C). Through these in vivo investigations, PHF20 may promote cSCC progression, potentially through modulation of PI3K/Akt signaling and DDR in association with GAS7 regulation. Furthermore, pan-squamous carcinoma survival analyses revealed cancer-type–specific prognostic associations in PHF20 and GAS7 [46]. In cervical as well as head and neck squamous carcinomas, high PHF20 tended to correlate with poorer survival, while GAS7 predicted favorable outcomes. In contrast, in esophageal squamous carcinoma, PHF20 expression unexpectedly correlated with improved survival (Fig. S7).Together with our cSCC data, these findings may unveil the PHF20–GAS7 axis serving as a clinically relevant regulatory module across squamous malignancies possibly, albeit with tumor-specific functional contexts.

## Discussion

Our study identifies PHF20 as a potential oncogenic regulator in cSCC and implicates it in the modulation of DDR. We describe a previously unrecognized pathway wherein PHF20 promotes the ubiquitin-mediated degradation of GAS7, leading to suppressed nuclear F-actin polymerization [44], ultimately, sensitization to genotoxic stress. This PHF20-GAS7-nuclear F-actin axis represents a potentially targetable vulnerability in advanced cSCC.

A key finding of our work is the repositioning of PHF20 from a classical epigenetic regulator to a direct modulator of nuclear architecture through its control of GAS7. The significance of nuclear actin dynamics in DDR is an emerging paradigm, with actin-binding proteins like JMY and Formin-2 known to facilitate repair by organizing F-actin networks [45, 48]. Our results position the PHF20-GAS7 duo as a critical rheostat in this process. By destabilizing GAS7, PHF20 curtails the formation of nuclear F-actin scaffolds, which have been reported to contribute to the spatial reorganization of chromatin and the assembly of repair complexes, such as 53BP1, BRCA2, RAD51, at DSB sites [18, 46], consistent with reports that nuclear F-actin also regulates PML nuclear bodies during DDR [49]. This provides a mechanistic link between a chromatin-associated reader and the physical mechanics of DNA repair.

As reported previously, by modulating the polymerization of nuclear F-actin, nuclear expression of GAS7 can provide the driving force for chromosomal movements during DDR [47, 50]. Beyond post-translational regulation, PHF20 may also suppress GAS7 transcriptionally. PHF20 has been revealed to interact with transcriptional complexes such as NSL and MLL [51, 52], and it can bind to the GAS7 promoter, as suggested by ChIP-seq data from public datasets [16]. Despite the absence of experimental confirmation, these data raise the possibility that PHF20 can suppress GAS7 in multiple layers to coordinate chromatin remodeling and DNA repair fidelity. Nevertheless, it should be acknowledged that there are still several limitations in our study. Firstly, while the IHC staining indicates an enrichment of PHF20 in cSCC tissues, further studies utilizing additional antibody clones or mass spectrometry-based proteomics would be beneficial to definitively map the subcellular partitioning of PHF20 in different clinical stages. Second, there is a lack of definition of the precise ubiquitination sites and associated E3 ligases, although our ubiquitination assays demonstrated that PHF20 could promote GAS7 degradation. Furthermore, even with the confirmation of PHF20 knockdown impairing tumor growth and enhancing apoptosis by our in vivo data, this study did not evaluate combined genotoxic therapies in animal models to assess therapeutic synergy.

In conclusion, we describe a PHF20–GAS7–nuclear F-actin pathway that contributes to the regulation of DDR fidelity. These findings suggest that this axis may represent a potential therapeutic strategy for advanced cSCC.

## Supplementary information

Supplementary information

checklist

Original western blots

## Data Availability

The data that support the findings of this study are available from the corresponding author upon reasonable request.
