# Supplementary material for: PHF20 stabilizes the GAS7-F-actin axis to drive DNA damage repair and chemoresistance in cutaneous squamous cell carcinoma
Source: Cell Death Dis. 2026 May 29;17(1):668. doi: 10.1038/s41419-026-08932-6 (PMC13424561; doi:10.1038/s41419-026-08932-6)
Supplement: Supplementary file 1 — Supplementary information [file 41419_2026_8932_MOESM1_ESM.docx]

Supplementary information

**Title: PHF20 stabilizes the GAS7-F-actin axis to drive DNA damage repair and chemoresistance in cutaneous squamous cell carcinoma**

Running title: PHF20-GAS7 Axis in Skin Cancer

Yunqian Li^1, 2, *^, He Wen^1, *^, Han Zheng^3^, Yunyue Zhen^1, 2^, Jing Jia^4^, Zhengjun Li^1^

^1^ Department of Dermatology, Qilu Hospital of Shandong University, Jinan, Shandong, 250012, P. R. China

^2^ Laboratory of Basic Medical Science, Qilu Hospital of Shandong University, Jinan, Shandong, 250012, P. R. China

^3^ Department of Orthopedics, The Second Qilu Hospital of Shandong University, Jinan, Shandong, 250012, P. R. China

^4^ Department of Pharmacy, Affiliated Hospital of Shandong University of Traditional Chinese Medicine, Jinan, Shandong, 250012, P. R. China

^*^ These authors contributed equally to this work.

**Corresponding author:** Zhengjun Li ([lzj19861126@163.com](mailto:lzj19861126@163.com)); Jing Jia ([imjiajing@163.com](mailto:imjiajing@163.com))

Supplementary Materials

Dataset Acquisition

Public gene expression profiles were retrieved from the GEO database (Table S1) [1, 2].

Table S1. Information of GEO datasets

| Dataset accession | Species | Number of samples | Platform | PMID |
| --- | --- | --- | --- | --- |
| GSE45216 | Human | 40 | GPL570 | 24335922 |
| GSE98767 | Human | 54 | GPL10558 |  |

RNA Extraction and Quantitative PCR

Total RNA from cells or tissues was isolated using TRIzol (Invitrogen) and reverse transcribed with the PrimeScript RT kit (Takara). qPCR was conducted using TB Green Premix (Takara) on a CFX96 Real-Time PCR system (Bio-Rad). Primer sequences are listed in Table S2.

Table S2. List of primers for qRT-PCR

| Primers | Forward sequence (5’-3’) | Reverse sequence (5’-3’) |
| --- | --- | --- |
| Human GAPDH | GTGAAGGTCGGAGTCAACGG | GCAACAATATCCACTTTACCAGAGT |
| Human PHF20 | ATGGCCTGCAGCTCAAGATGA | CTCCCCTGAGTGCTGTTTCCA |
| Human GAS7 | GCAGAGCAAGGAAAACACCA | GGTCCTTCTTATCAGCCCAG |

Western Blotting

Proteins were extracted with RIPA buffer (Beyotime) and quantified using the BCA assay. After SDS‒PAGE and PVDF membrane transfer (Millipore), membranes were blocked and incubated with specific primary antibodies (1:1000, overnight, 4 °C), followed by secondary antibody (1:2000) incubation. Signals were visualized using ECL Plus (Millipore) and detected with the e-BLOT imaging system. Antibodies are detailed in Table S3.

Table S3. List of antibody information

| Antibody | Clone | Distributor | Dilution |
| --- | --- | --- | --- |
| PHF20 | #3934 | Cell Signaling Technology, Boston, USA | WB:1/1000  Co-IP:1/50 |
| PHF20 | 22010-1-AP | Proteintech, Rosemont, IL, USA | WB:1/1000  IF:1/100 |
| PHF20 | IHC-00698 | Thermo Fisher Scientific, Waltham, MA, USA | IHC:1/250 |
| GAS7 | sc-365385 | Santa Cruz Biotechnology, Dallas, TX, USA | Co-IP:1/50 |
| GAS7 | 10072-1-AP | Proteintech, Rosemont, IL, USA | WB:1/1000  IF:1/200 |
| GAPDH | #97166 | Cell Signaling Technology, Boston, USA | WB:1/1000 |
| Histone3 | #4499 | Cell Signaling Technology, Boston, USA | WB:1/2000 |
| β-Actin | EM21002 | Huabio, Hangzhou, China | WB:1/80000 |
| cyclin-D1 | #2978 | Cell Signaling Technology, Boston, USA | WB:1/1000 |
| cyclin-E1 | #20808 | Cell Signaling Technology, Boston, USA | WB:1/1000 |
| N-cadherin | 66219-1-Ig | Proteintech, Rosemont, IL, USA | WB:1/5000 |
| E-cadherin | 60335-1-Ig | Proteintech, Rosemont, IL, USA | WB:1/5000 |
| Vimentin | 60330-1-Ig | Proteintech, Rosemont, IL, USA | WB:1/50000 |
| MMP2 | 66366-1-Ig | Proteintech, Rosemont, IL, USA | WB:1/2000 |
| MMP9 | 10375-2-AP | Proteintech, Rosemont, IL, USA | WB:1/1000 |
| p53 | Ab26 | Abcam, Cambridge, UK | WB:1/1000 |
| Bcl-2 | ET1702-53 | Huabio, Hangzhou, China | WB:1/5000 |
| Bax | ET1603-34 | Huabio, Hangzhou, China | WB:1/20000 |
| p-γ-H2AX | AB3322 | Abways Technology, Shanghai, China | WB:1/1000  IF:1/100 |
| p-PI3K | CY6428 | Abways Technology, Shanghai, China | WB:1/1000 |
| p-Akt | CY5885 | Abways Technology, Shanghai, China | WB:1/2000  IF:1/30 |
| PI3K | CY6915 | Abways Technology, Shanghai, China | WB:1/2000 |
| Akt | #9272 | Cell Signaling Technology, Boston, USA | WB:1/1000 |
| Caspase3 | #9662 | Cell Signaling Technology, Boston, USA | WB:1/1000 |
| cleaved-Caspase3 | #9661 | Cell Signaling Technology, Boston, USA | WB:1/1000 |
| Caspase7 | #9492 | Cell Signaling Technology, Boston, USA | WB:1/1000 |
| cleaved-Caspase7 | #8438 | Cell Signaling Technology, Boston, USA | WB:1/1000 |

Cell Proliferation Assays

Cell viability was assessed using the CCK-8 kits at 6, 24, 48, and 72 h post-transfection, with absorbance measured at 450 nm (Tecan Infinite M200). EdU incorporation assays (RiboBio, Guangdong, China) were used to visualize DNA synthesis. EdU-labeled cells were fixed, permeabilized, and stained with DAPI; fluorescence images were acquired for quantification.

Wound Healing Assay

Confluent monolayers in 6-well plates were scratched using sterile pipette tips. After PBS washing, cells were cultured in serum-reduced medium. Wound areas were photographed at 0 h and subsequent time points; migration was quantified by calculating wound closure percentages.

Transwell Migration and Invasion Assays

Transwell chambers (8 μm pore, Millipore, Burlington, MA, USA) were used to assess migration and invasion. For migration, 1×10⁵ cells in serum-free medium were placed in upper chambers, with 10% FBS medium below. After 48 h, migrated cells were fixed, stained with crystal violet, and counted. For invasion, chambers were pre-coated with Matrigel, and 2×10⁵ cells were seeded under the same conditions.

Cell Cycle Analysis and Apoptosis Detection

Cells were fixed in 70% ethanol at 4 °C for 2 h, treated with RNase A, and stained with PI (Solarbio, Beijing, China). DNA content was analyzed using a FACSCalibur flow cytometer (BD Biosciences). Apoptosis was evaluated using Annexin V/PI staining (Solarbio, Beijing, China). After 48 h post-transfection, cells were stained in the dark and analyzed by flow cytometry (FACSCalibur, BD Biosciences).

TUNEL Assay

Cells were cultured on coverslips and treated with siRNA for 48 hours. After fixation with 4% paraformaldehyde, cells were permeabilized using 0.1% Triton X-100 and incubated with the TUNEL reaction mixture (Beyotime, China) at 37°C for 1 hour in the dark. Slides were counterstained with DAPI, mounted, and imaged under a fluorescence microscope. TUNEL-positive cells were quantified to assess apoptosis levels.

Micronucleus Assay

To evaluate genomic instability, micronucleus formation was assessed as previously described [3, 4]. Post-siRNA transfection, cells were exposed to bleomycin (4 μg/mL) for 2 hours, followed by cytochalasin B (6 μg/mL) for 48 hours. Cells were then harvested, subjected to hypotonic treatment (0.075 M KCl, 20 min), and stained with acridine orange (0.01%). Approximately 1,000 binucleated cells per group were analyzed under a fluorescence microscope to determine micronucleus frequency. Micronuclei were identified according to established morphological criteria: round or oval extranuclear bodies with a diameter less than one-third of the main nucleus, clearly separated from but within the cytoplasm of binucleated cells, and displaying similar staining intensity to the main nuclei. Apoptotic bodies and nuclear fragments were excluded from the analysis. Assays were performed in biological triplicates.

Immunohistochemistry (IHC) and Immunofluorescence (IF)

Paraffin-embedded tissues were processed via standard protocols, including deparaffinization, rehydration, antigen retrieval (citrate buffer, pH 6.0), and blocking with serum. Sections were incubated with primary antibodies overnight at 4°C, followed by HRP-conjugated or fluorescently labeled secondary antibodies for IHC and IF, respectively. DAB was used for IHC signal development, and DAPI was used for nuclear counterstaining in IF. Fluorescence signals were visualized with an Olympus microscope. For IF on cells, A431 and SCL-1 lines were seeded on coverslips, fixed, permeabilized, and subjected to the same staining procedure. Quantification was performed using ImageJ. Antibodies are listed in Table S3.

Multiplex Immunohistochemistry (mIHC)

Cells grown on coverslips were fixed with paraformaldehyde, permeabilized, and blocked as above. A mixture of primary antibodies was applied overnight at 4°C. After secondary antibody incubation and signal amplification using TSA (1:100), the procedure was repeated sequentially for multiplex labeling. Finally, nuclei were stained with DAPI, and slides were mounted and imaged on a ZEISS fluorescence microscope. Signal quantification was conducted via ImageJ.

RNA-Seq and Data Analysis

Total RNA was extracted from A431 and SCL-1 cells 48 h after siRNA treatment using the RNeasy Mini Kit (Qiagen). RNA quality was verified on an Agilent 2100 Bioanalyzer. Libraries were prepared using the TruSeq RNA Sample Prep Kit (Illumina), and sequencing was performed on the Illumina NovaSeq platform. Raw reads were mapped to the GRCh37/hg19 genome using STAR, and gene counts were generated using FeatureCounts. Differential expression was analyzed using DESeq2. Gene Ontology and KEGG pathway enrichment were conducted using DAVID and clusterProfiler. The RNA-sequencing data generated in this study have been deposited in the Genome Sequence Archive (GSA) under accession number PRJCA046937 (https://www.cncb.ac.cn).

References

[1] Lambert SR, Mladkova N, Gulati A, Hamoudi R, Purdie K, Cerio R, *et al*. Key differences identified between actinic keratosis and cutaneous squamous cell carcinoma by transcriptome profiling. Br J Cancer. 2014;110(2):520-529.

[2] Inman GJ, Wang J, Nagano A, Alexandrov LB, Purdie KJ, Taylor RG, *et al*. The genomic landscape of cutaneous SCC reveals drivers and a novel azathioprine associated mutational signature. Nat Commun. 2018;9:3667.

[3] Huang M, Zhou B, Gong J, Xing L, Ma X, Wang F, *et al*. RNA-splicing factor SART3 regulates translesion DNA synthesis. Nucleic Acids Res. 2018;46(9):4560-4574.

[4] Mansilla SF, Soria G, Vallerga MB, Habif M, Martínez-López W, Prives C, *et al*. UV-triggered p21 degradation facilitates damaged-DNA replication and preserves genomic stability. Nucleic Acids Res. 2013;41(14):6942-6951.

Supplementary Figure legends


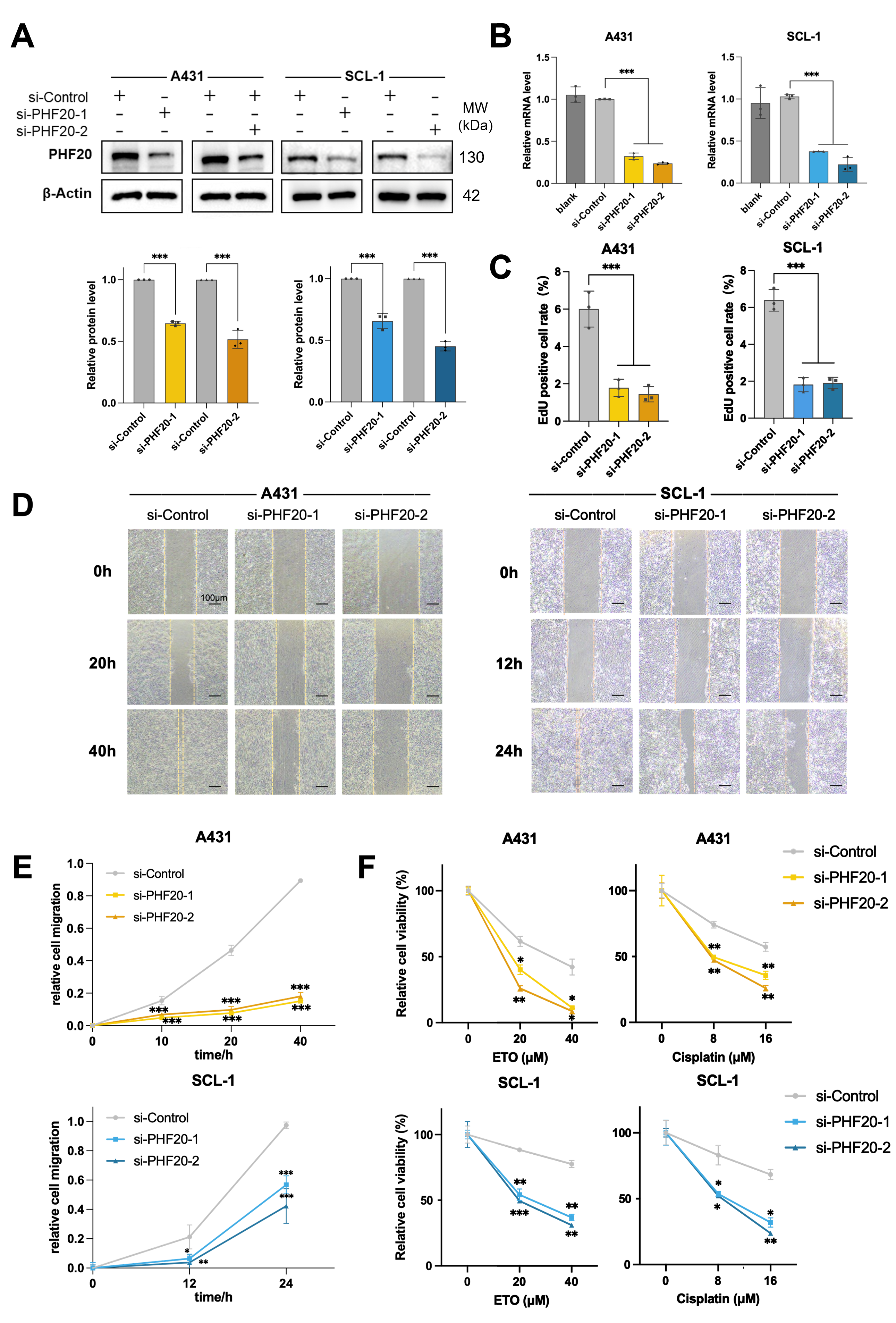


**Figure S1. Efficiency of PHF20 knockdown and quantification of EdU.**

**A** Western blot analysis of PHF20 after PHF20 knockdown. **B** The efficiency of PHF20 knockdown was measured by qRT‒PCR. **C** Cell proliferation was assessed by EdU incorporation assay following PHF20 knockdown. **D, E** Wound healing assay and quantification of PHF20 depletion cSCC cell migration ability. Bar=100 μm. **F** Sensitivity assays of PHF20-depleted cells treated with the indicated doses of ETO and cisplatin for 48 hours. Data are shown as mean ± SD from three independent experiments. ***p<0.001.


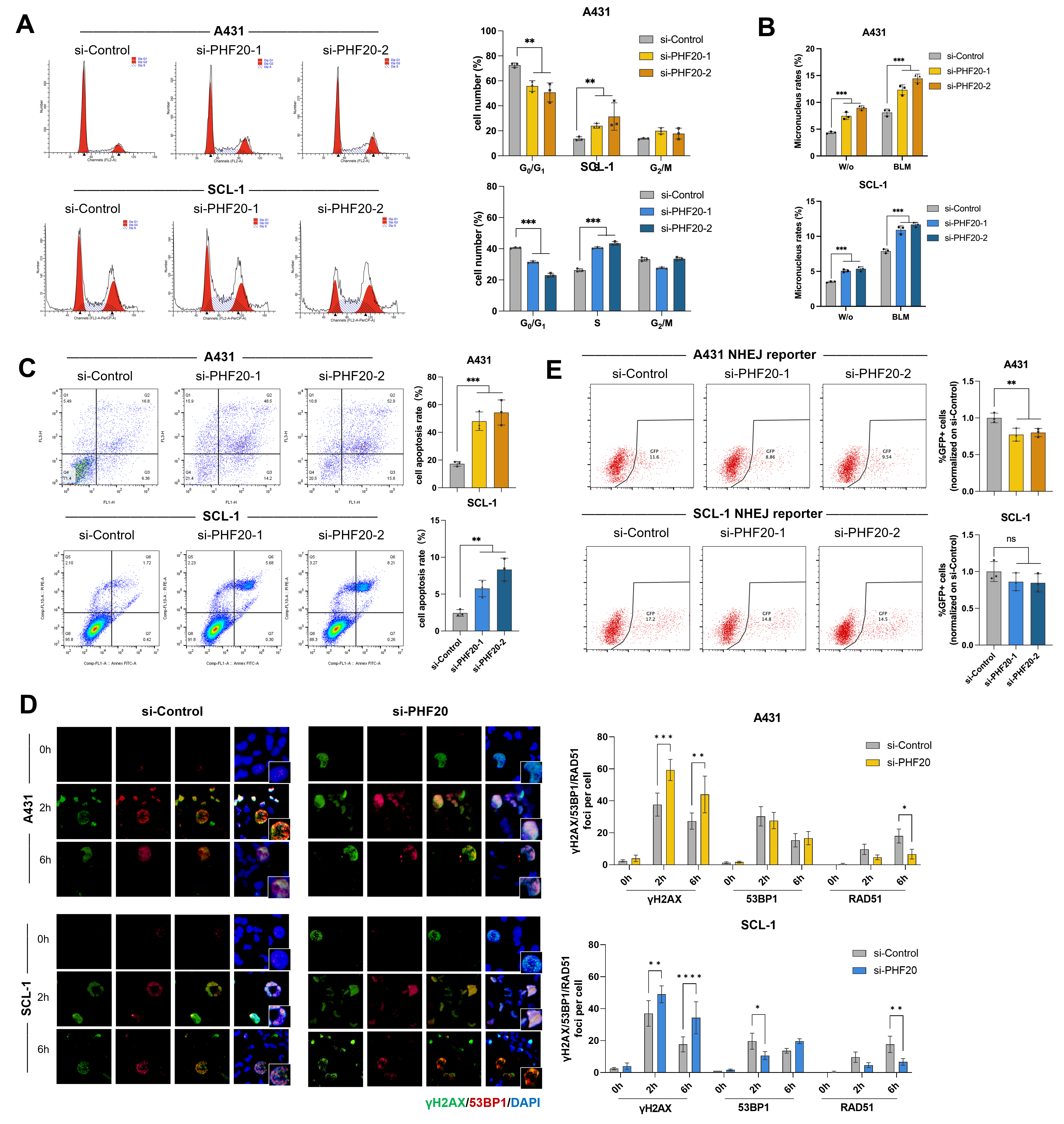


**Figure S2. PHF20 loss induces replication stress, genomic instability and apoptosis.**

**A** Flow cytometry analysis of the cell cycle and quantification at 48 hours after PHF20 depletion. **B** Percentage of PHF20-depleted cSCC cells containing one or more micronuclei after treatment with bleomycin (4 μg/ml) for 2 hours. **C** Flow cytometry analysis of apoptosis and quantification at 48 hours after PHF20 depletion. **D** Representative immunofluorescence analysis of γH2AX and 53BP1 foci formation following DNA damage. Bar=10 μm. **E** Quantification of NHEJ repair efficiency using EJ5-GFP reporter assays. Data are shown as mean ± SD from three independent experiments. ***p<0.001.


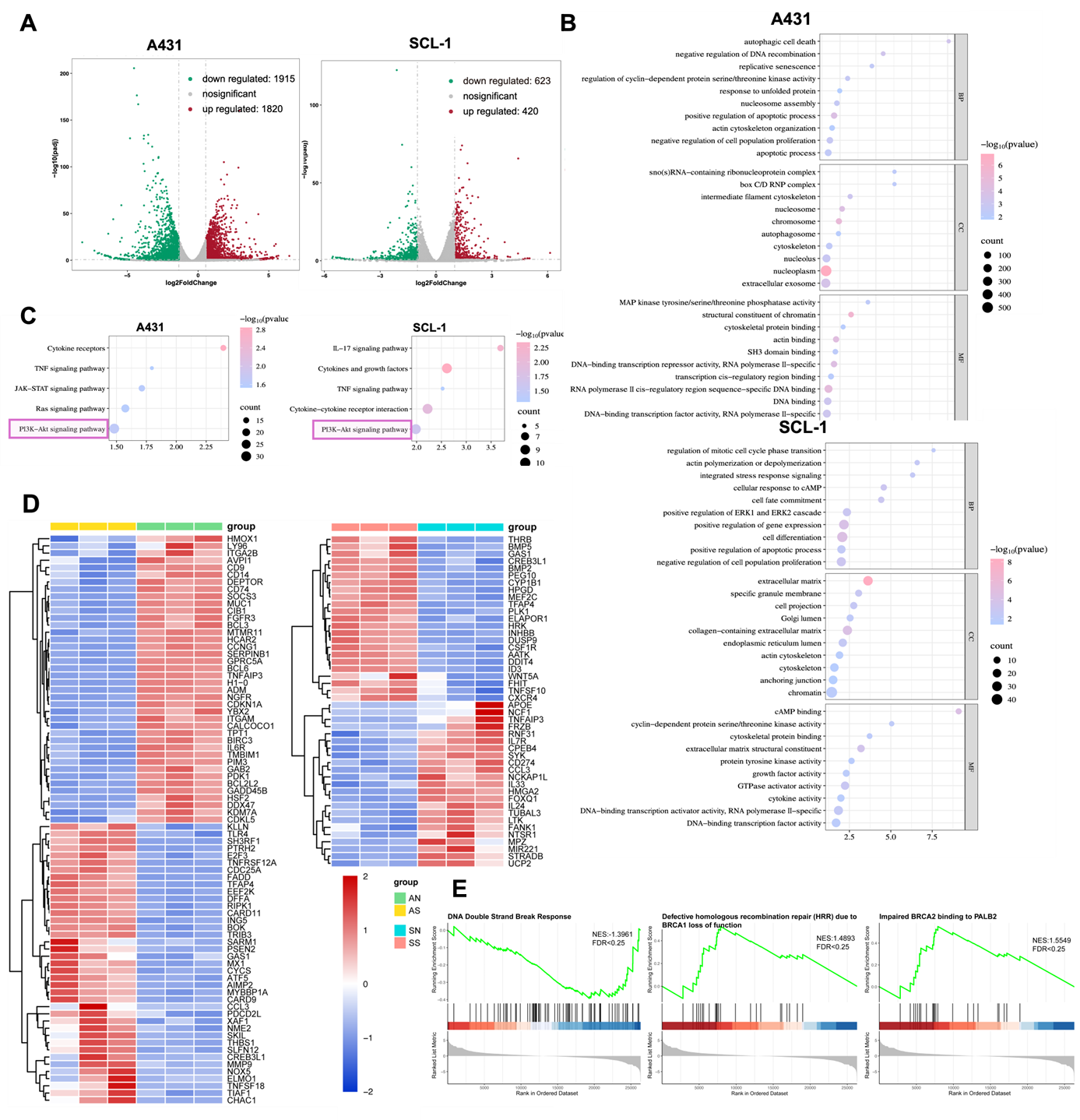


**Figure S3. Transcriptomic and pathway analysis after PHF20 knockdown.**

**A** Volcano plot showing differentially expressed genes (DEGs) identified by RNA-seq following PHF20 knockdown. **B** GO enrichment analysis of all DEGs.KEGG pathway enrichment analysis of all DEGs. **C** KEGG pathway enrichment analysis of downregulating DEGs. **D** Heatmap of apoptosis-related genes according to the RNA-seq data. **E** GSEA revealed enriched signaling pathways in the RNA-seq data of PHF20-depleted cells.


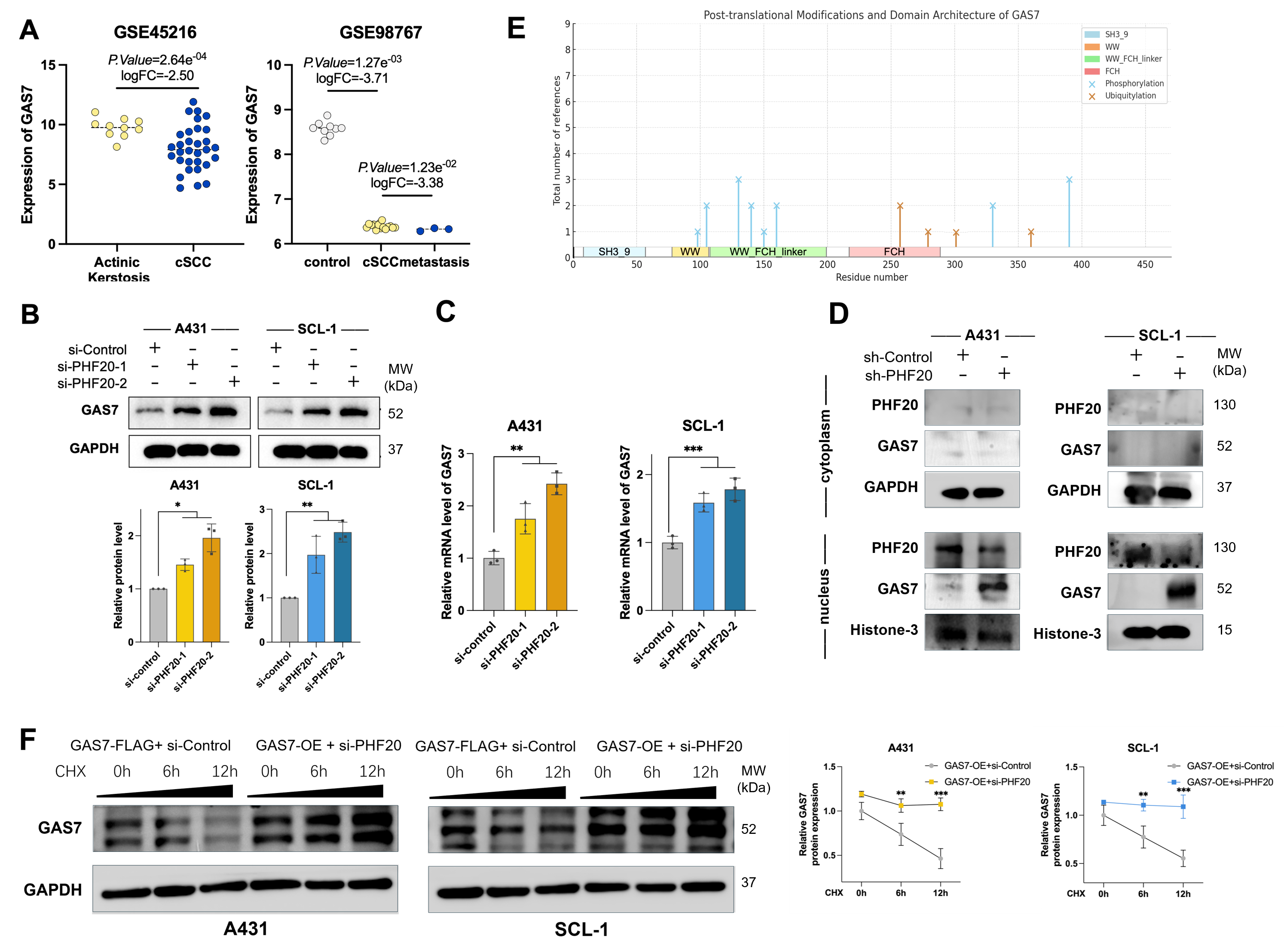


**Figure S4.** **PHF20 negatively regulates GAS7 expression and nuclear localization.**

**A** Differential expression analysis of GAS7 in cSCC tissues versus normal controls in GSE45216 and GSE98767. **B** Western blot analysis showing upregulation of GAS7 protein levels following PHF20 knockdown. **C** qRT-PCR analysis showing increased GAS7 mRNA levels after PHF20 knockdown. **D** Western blot analysis of GAS7 expression in the cytoplasmic and nuclear fractions of A431 and SCL-1 cells after PHF20 knockdown. GAPDH and histone-3 served as cytoplasmic and nuclear markers, respectively. **E** Distribution of predicted phosphorylation (blue) and ubiquitination (brown) sites across the GAS7 protein, aligned with its domain structure in PhosphoSitePlus. **F** Representative Western blot of GAS7 in A431 and SCL-1 cells ectopically expressing GAS7 with or without PHF20 and treated with cycloheximide (CHX) for the indicated time points are shown (left). Degradation curves of FLAG protein (right). Data are shown as mean ± SD from three independent experiments. *p<0.05, **p<0.01, and ***p<0.001.


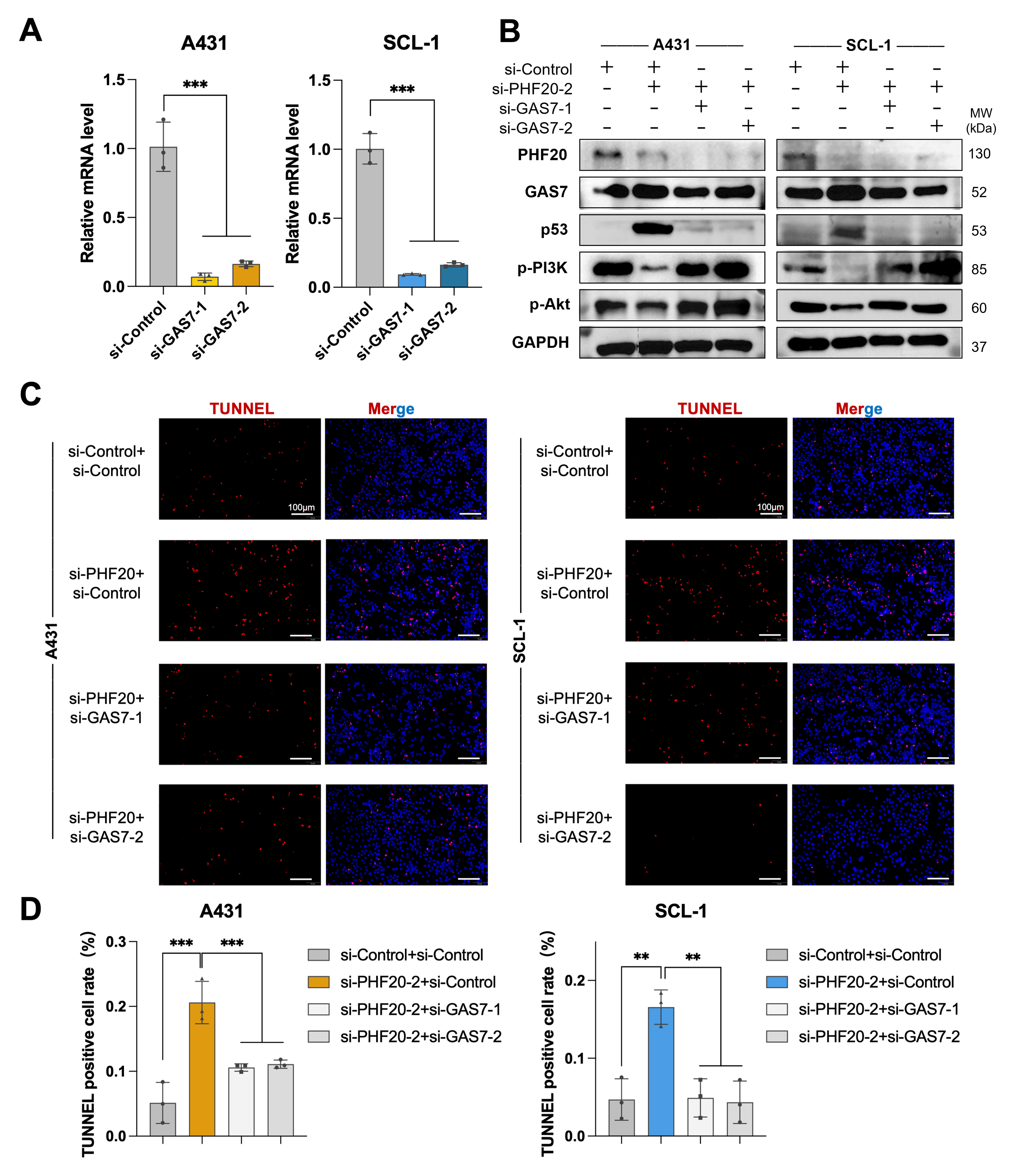


**Figure S5. GAS7 mediates the pro-apoptotic and signaling effects of PHF20 depletion in cSCC cells.**

**A** Validation of GAS7 knockdown efficiency by qRT-PCR in A431 and SCL-1 cells. **B** Western blot analysis of p53 and PI3K/Akt pathway activity in cSCC cells upon PHF20 knockdown alone or combined with GAS7 knockdown. **C** Representative fluorescence images of the TUNNEL (red) assay in PHF20-depleted cSCC cells with or without GAS7 depletion. Nuclei were stained with DAPI (blue), and a combined reaction involving TUNEL and DAPI indicated the number of damaged cells. Bar=100 μm. **D** Quantification of TUNEL-positive cells following PHF20 knockdown, with or without GAS7 co-depletion. Data are shown as mean ± SD from three independent experiments. **p<0.01, and ***p<0.001.


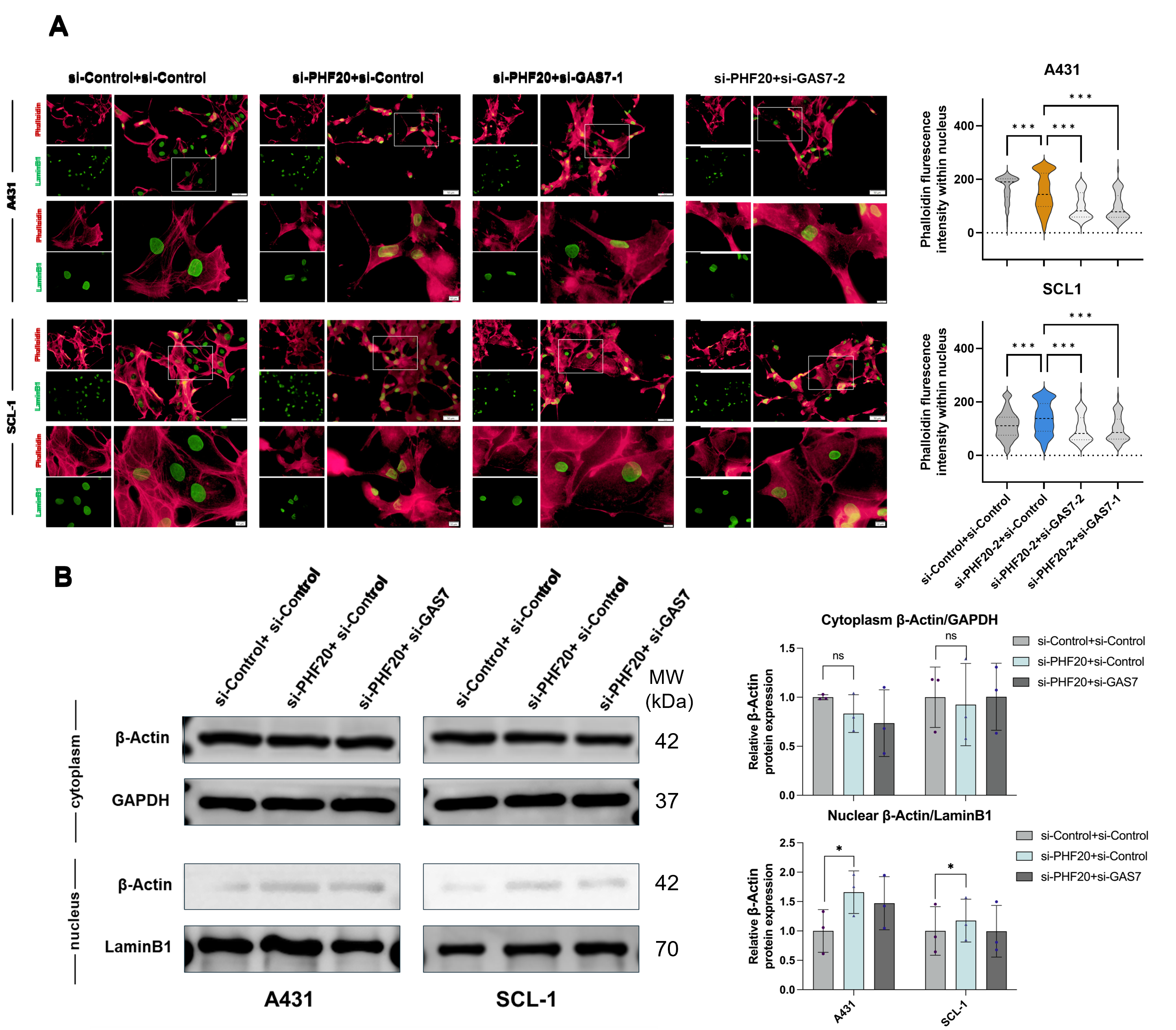


**Figure S6. PHF20 regulates nuclear F-actin accumulation in a GAS7-dependent manner.**

**A** Quantification of phalloidin fluorescence intensity within DAPI-defined nuclear regions. Data were obtained from three independent experiments, with at least 100 cells quantified per condition in each experiment. **B** Western blot analysis of β-actin in nuclear and cytoplasmic fractions after PHF20 depletion with or without GAS7 depletion. The data are shown as the means ± SDs from three independent experiments. **p<0.01, and ***p<0.001.


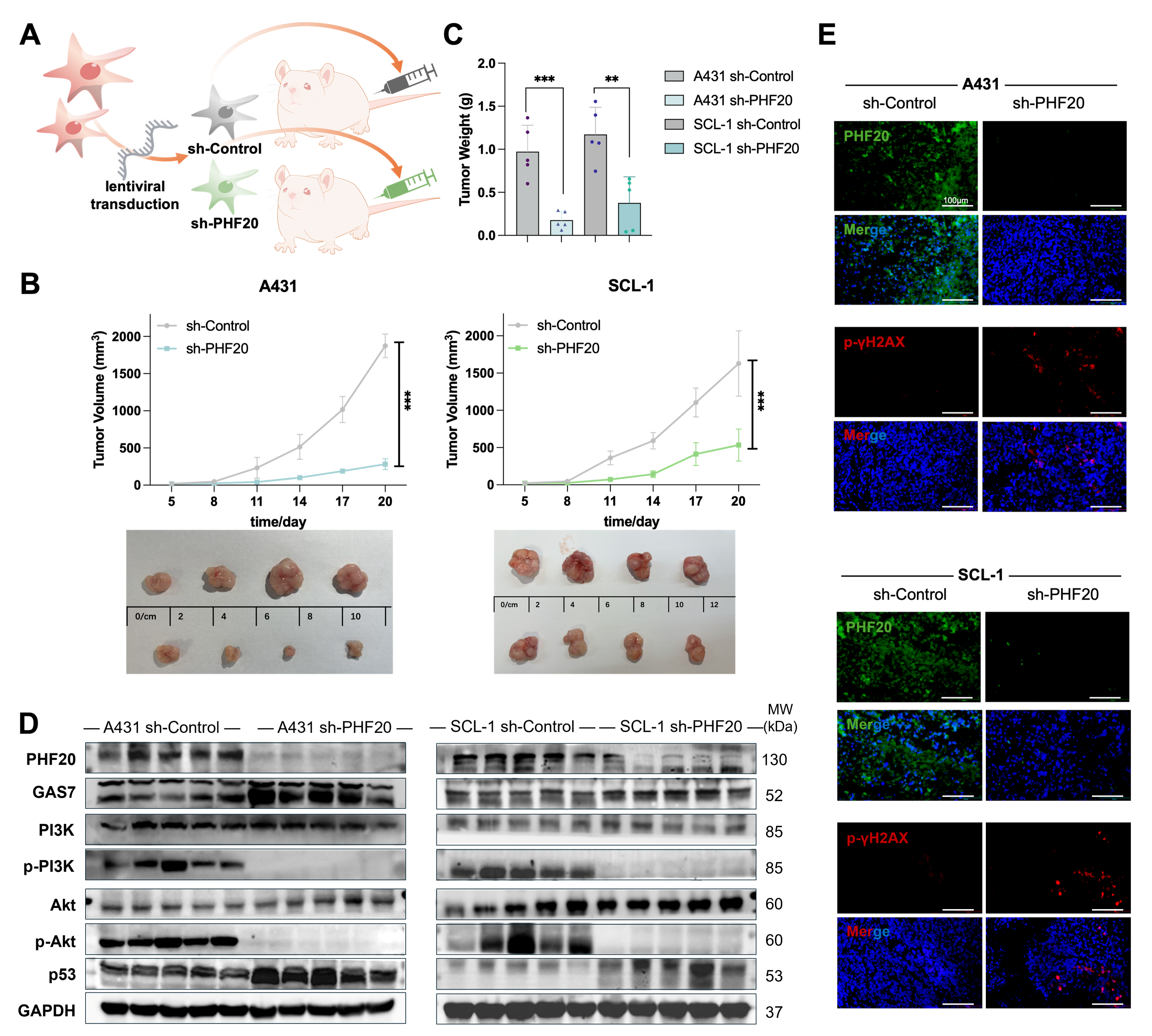


**Figure S7.** **PHF20 depletion suppresses cSCC tumor growth and modulates apoptosis-related signaling in vivo.**

**A** Representative images of xenograft tumors derived from PHF20-depleted and control cSCC cells. **B** Western blot analysis of apoptosis-related markers in xenograft tissues. **C** Immunofluorescence analysis of GAS7 and p-Akt in tumor sections.


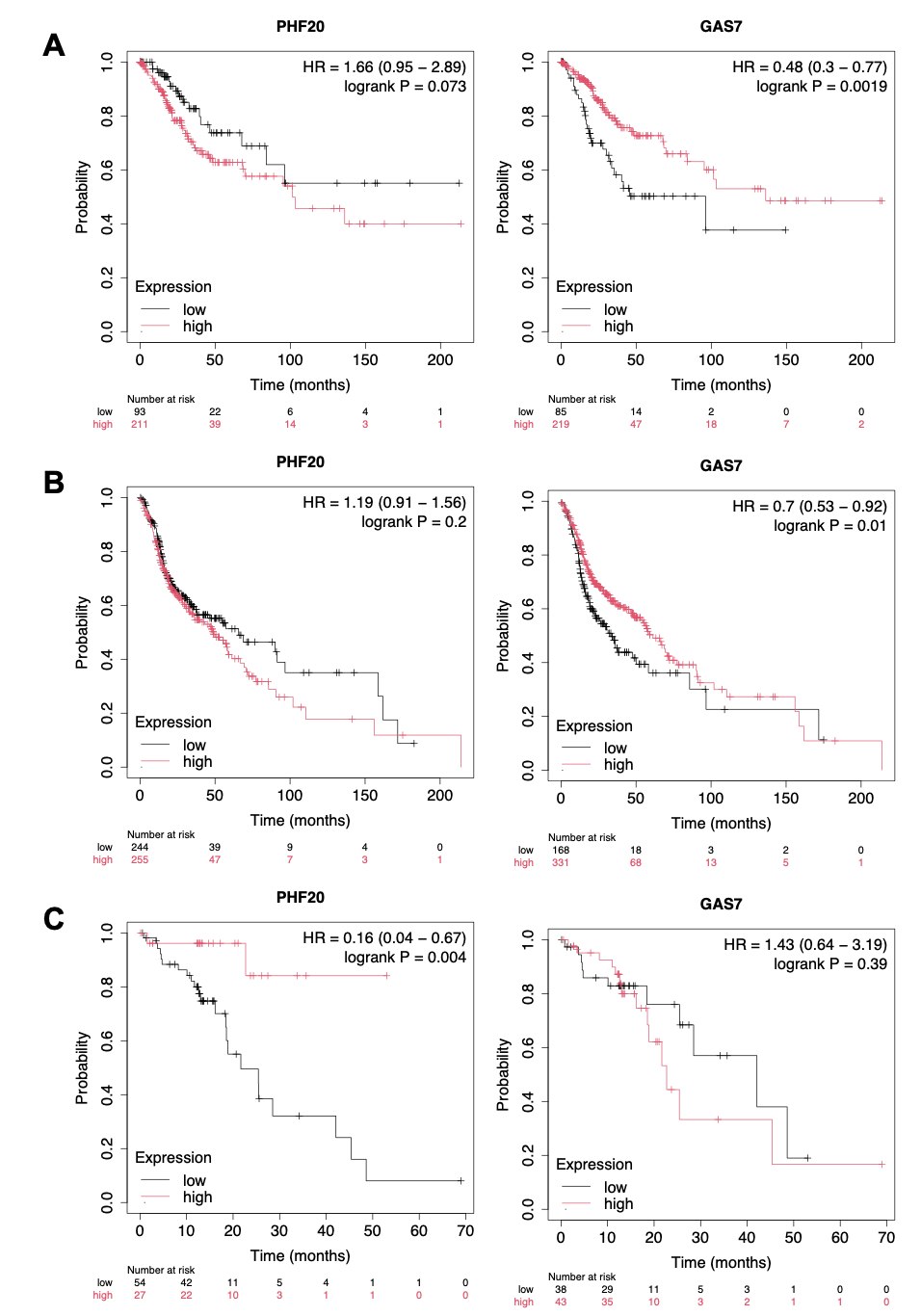


**Figure S8. Prognostic relevance of PHF20 and GAS7 expression across pan-squamous carcinomas.**

**A** Kaplan–Meier survival analysis of cervical squamous carcinoma patients stratified by PHF20 or GAS7 expression. **B** Kaplan–Meier survival analysis of head and neck squamous carcinoma patients stratified by PHF20 or GAS7 expression. **C** Kaplan–Meier survival analysis of esophageal squamous carcinoma patients stratified by PHF20 or GAS7 expression.
