# Supplementary material for: PHF20 stabilizes the GAS7-F-actin axis to drive DNA damage repair and chemoresistance in cutaneous squamous cell carcinoma
Source: Cell Death Dis. 2026 May 29;17(1):668. doi: 10.1038/s41419-026-08932-6 (PMC13424561; doi:10.1038/s41419-026-08932-6)

SourceData Figure 1E

PHF20

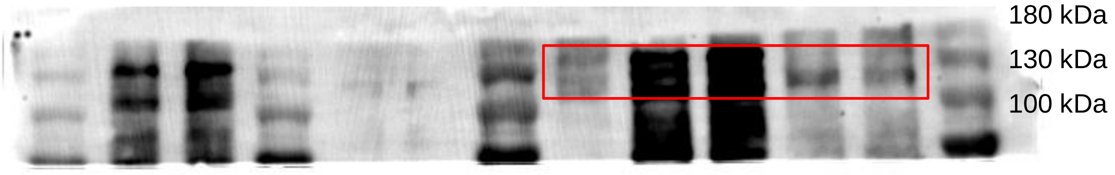

GAPDH

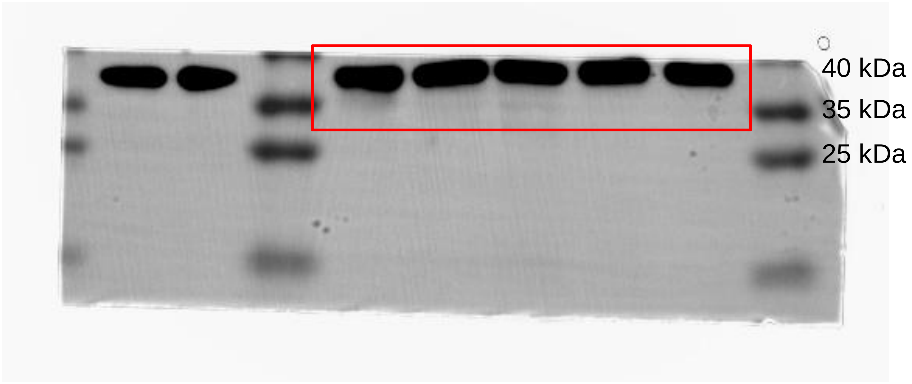

SourceData Figure 2C

A431-PHF20

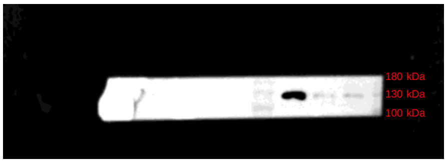

SCL-1-PHF20

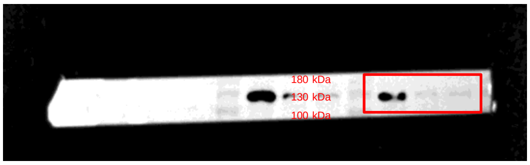

A431-cyclinD1

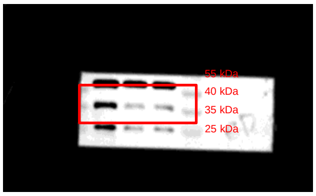

SCL-1-cyclinD1

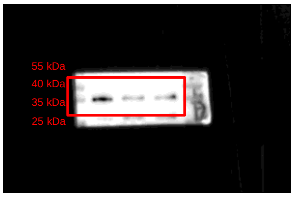

A431-cyclinE1

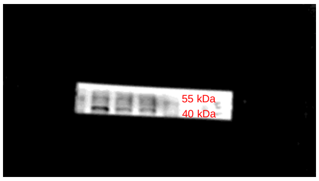

SCL-1-cyclinE1

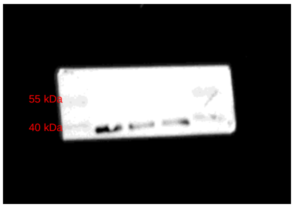

A431-MMP9

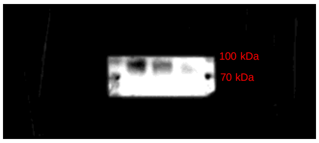

SCL-1-MMP9

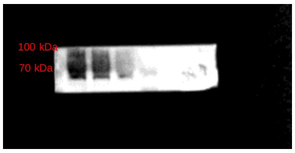

A431-MMP2

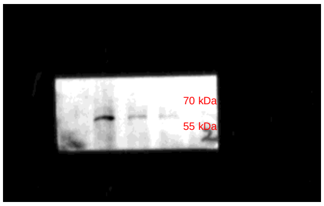

SCL-1-MMP2

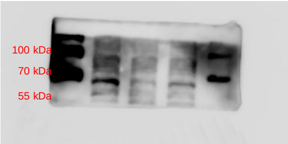

A431-N-cadherin

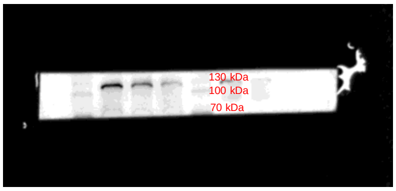

SCL-1-N-cadherin

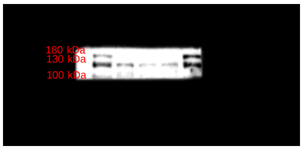

A431-E-cadherin

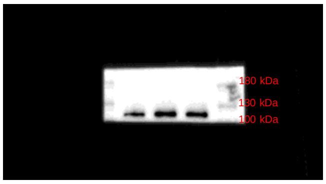

SCL-1-E-cadherin

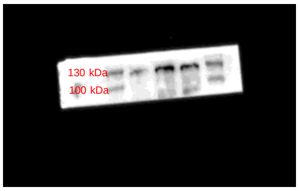

A431-Vimentin

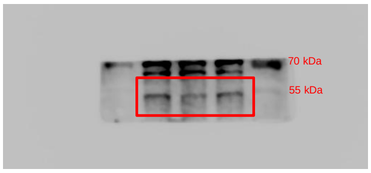

SCL-1-Vimentin

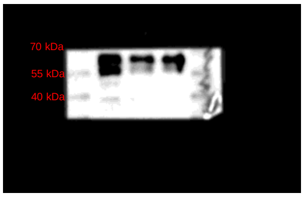

A431-β-Actin

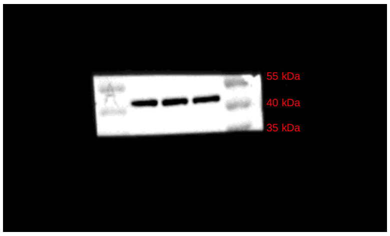

SCL-1-β-Actin

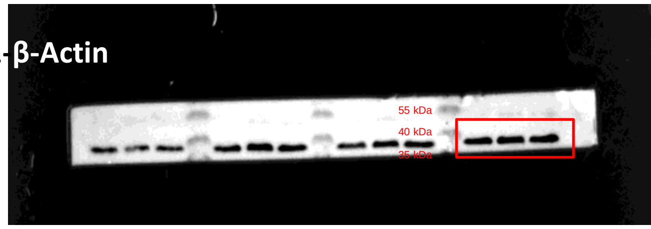

SourceData Figure 3A

A431-p53

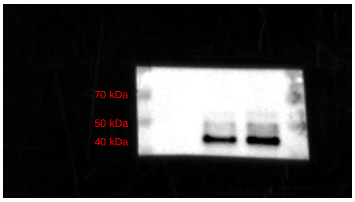

SCL-1-p53

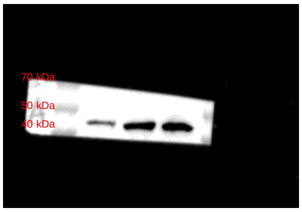

A431-Bcl-2

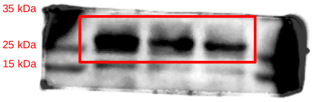

SCL-1-Bcl-2

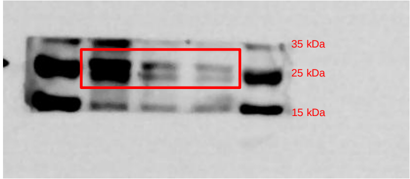

A431-Bax

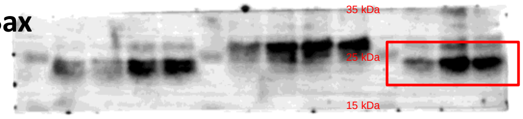

SCL-1-Bax

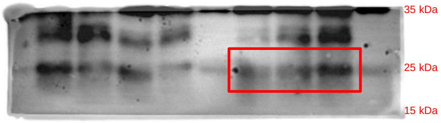

A431-GAPDH

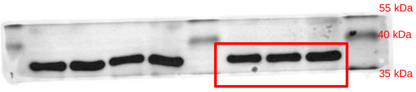

SCL-1-GAPDH

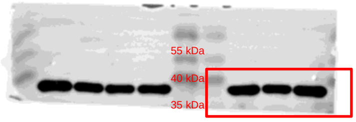

SourceData Figure 3C-A431

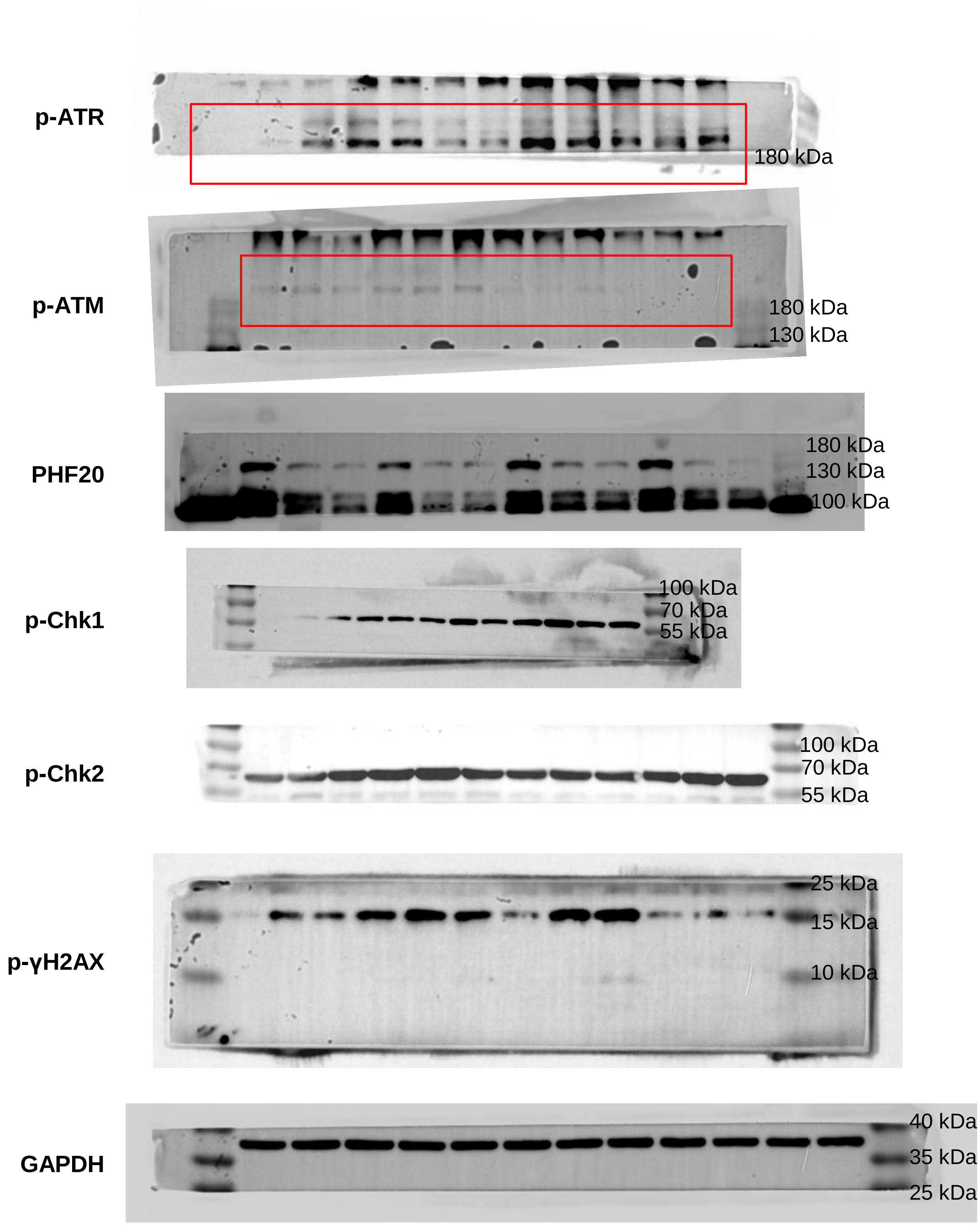

SourceData Figure 3C-SCL-1

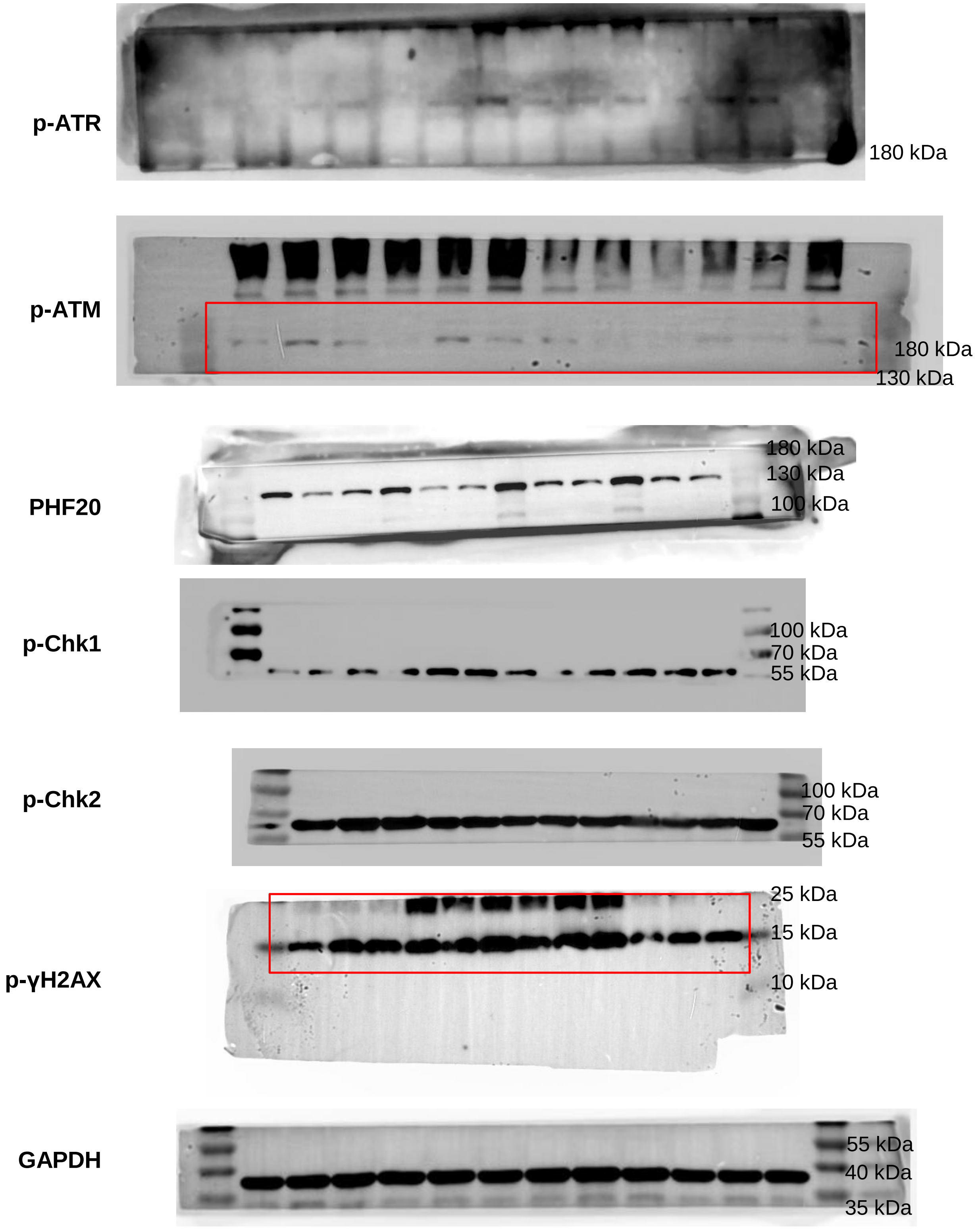

SourceData Figure 4C

IP: PHF20

A431-PHF20

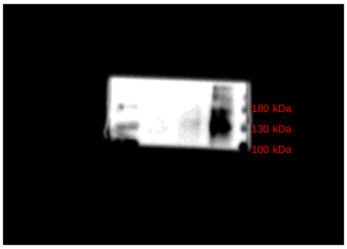

SCL-1-PHD20

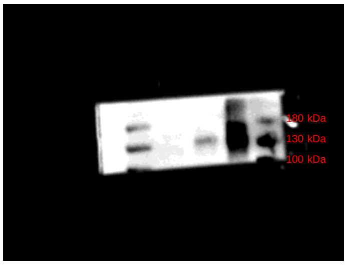

A431-GAS7

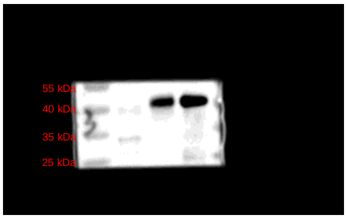

SCL-1-GAS7

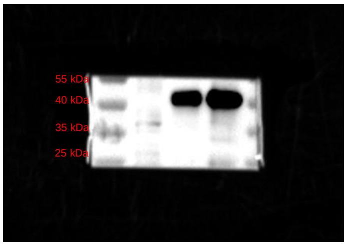

IP: GAS7

A431-PHF20

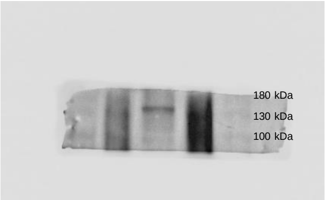

SCL-1-PHD20

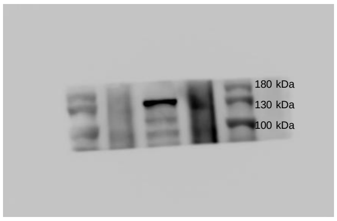

A431-GAS7

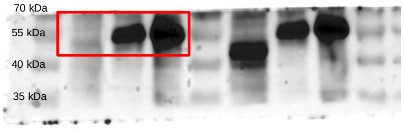

SCL-1-GAS7

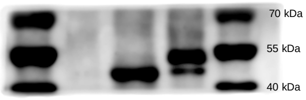

SourceData Figure 4D

A431-GAS7

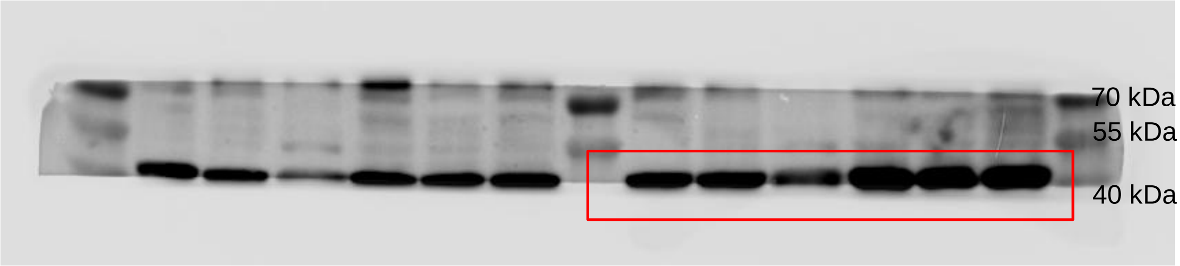

A431-GAPDH

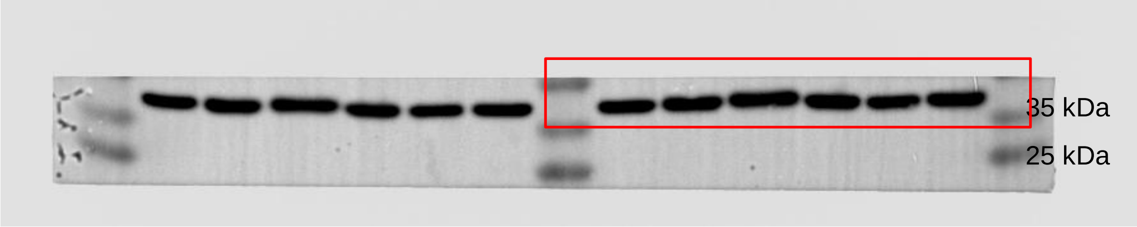

SCL-1-GAS7

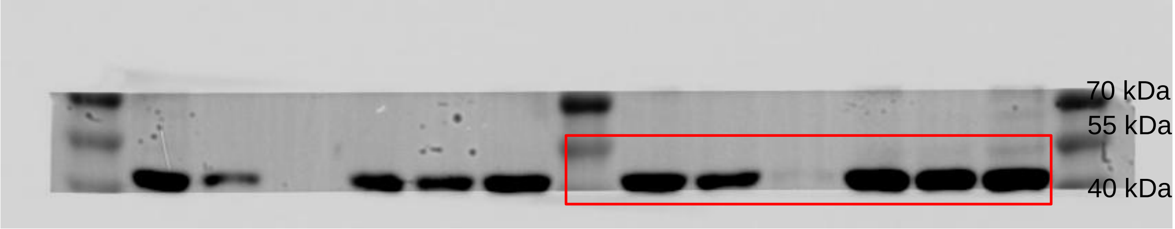

SCL-1-GAPDH

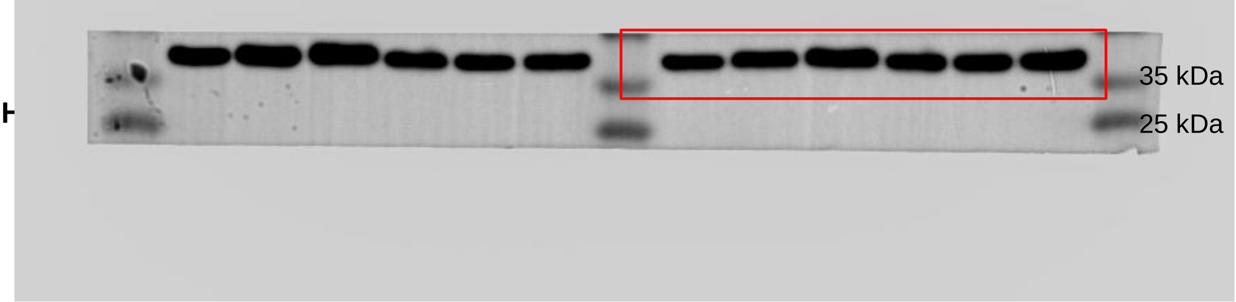

SourceData Figure 4E

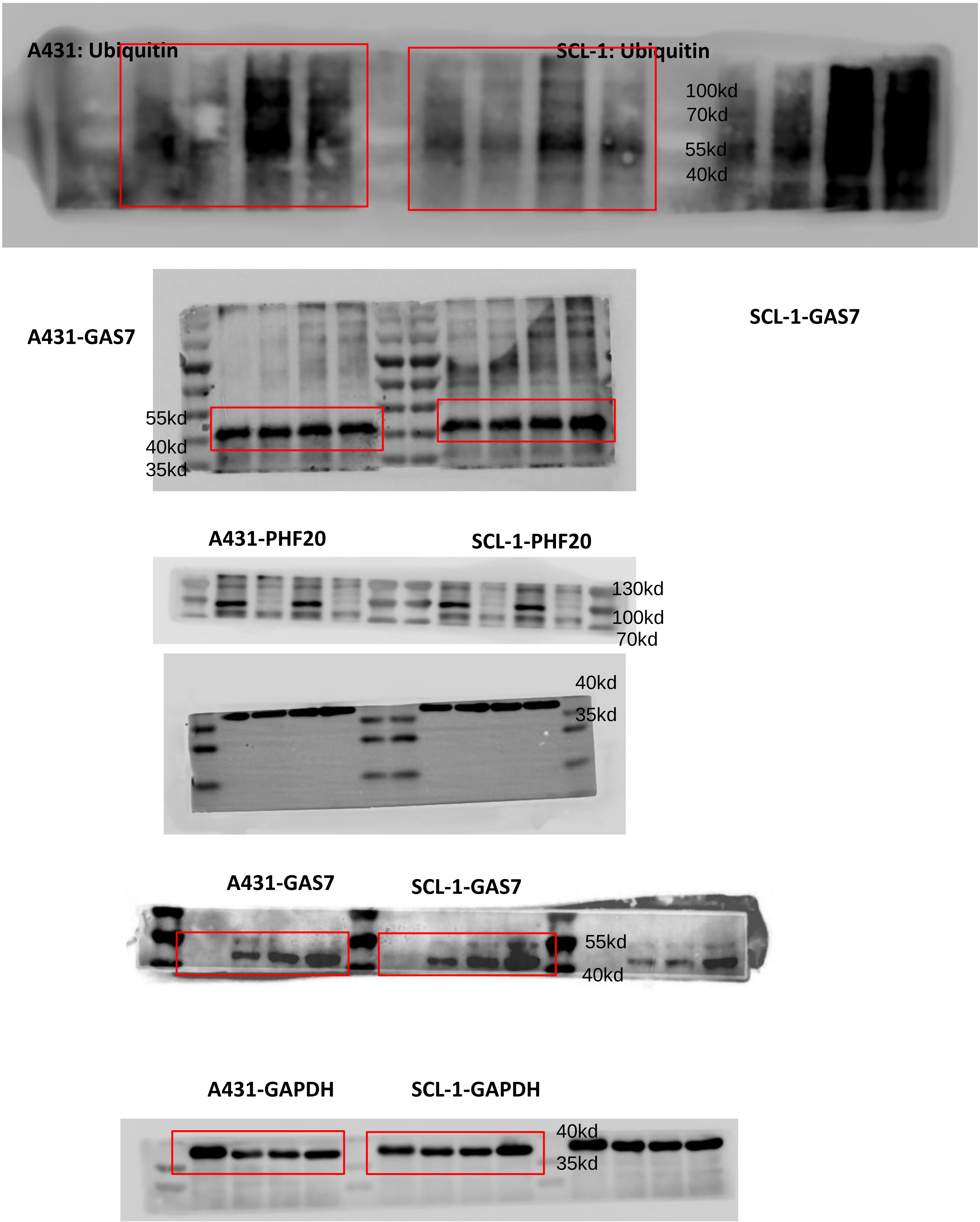

SourceData Figure 6D

A431-PHF20

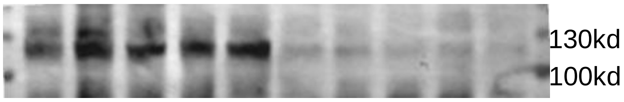

A431-GAS7

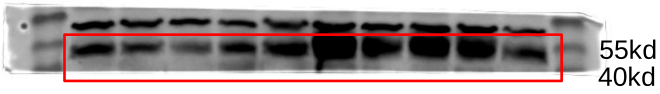

A431-PI3K

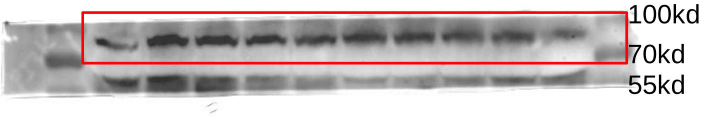

A431-p-PI3K

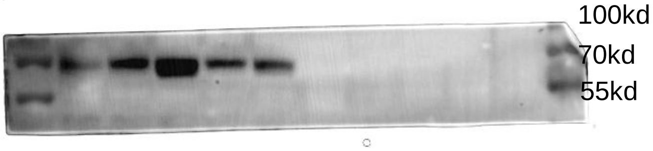

A431-Akt

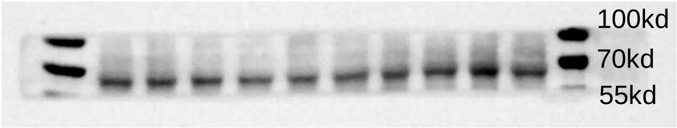

A431-p-Akt

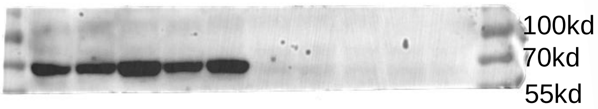

A431-p53

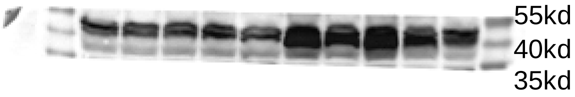

A431-GADPH

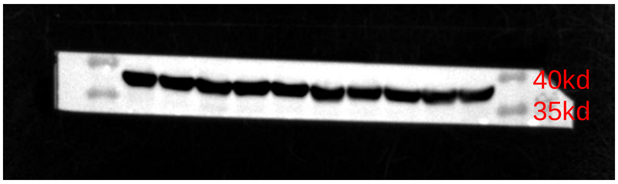

SCL-1-PHF20

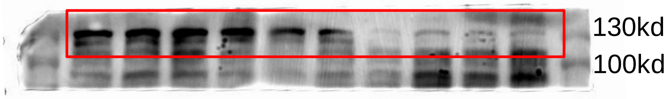

SCL-1-GAS7

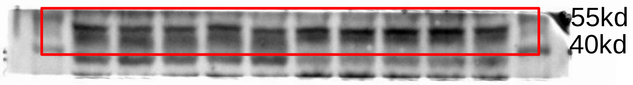

SCL-1-PI3K

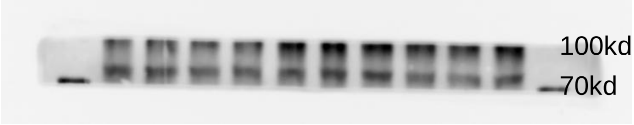

SCL-1-p-PI3K

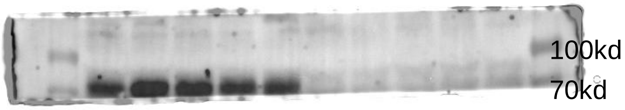

SCL-1-Akt

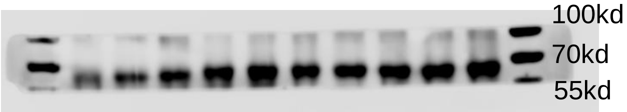

SCL-1-p-Akt

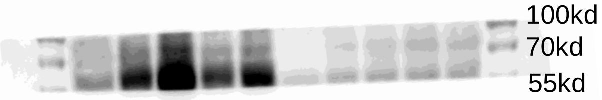

SCL-1-p53

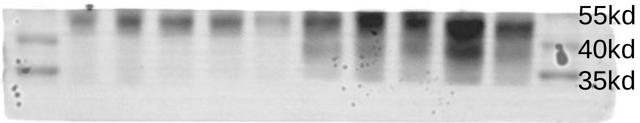

SCL-1-GADPH

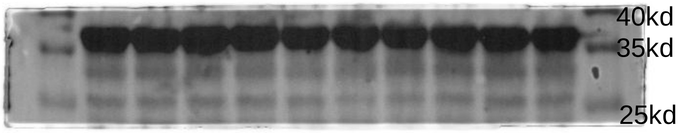

SourceData Figure S1A

A431

PHF20

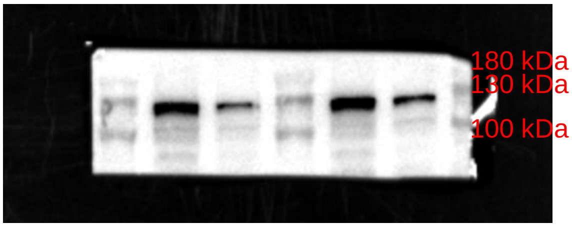

GAPDH

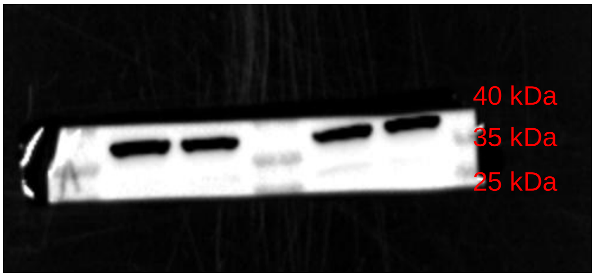

SCL-1

PHF20

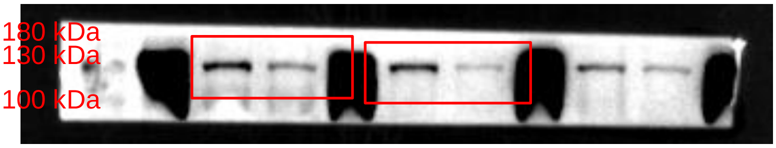

GAPDH

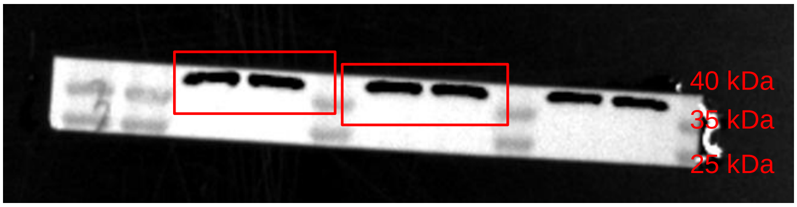

SourceData Figure S4B

A431 SCL-1

GAS7

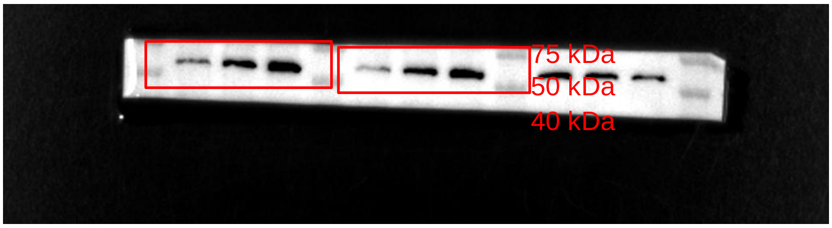

β-Actin

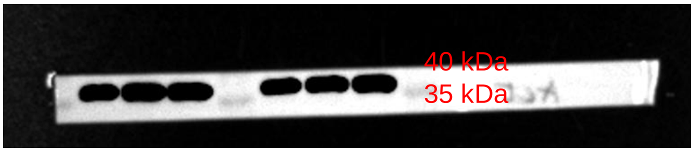

SourceData Figure S4D

A431

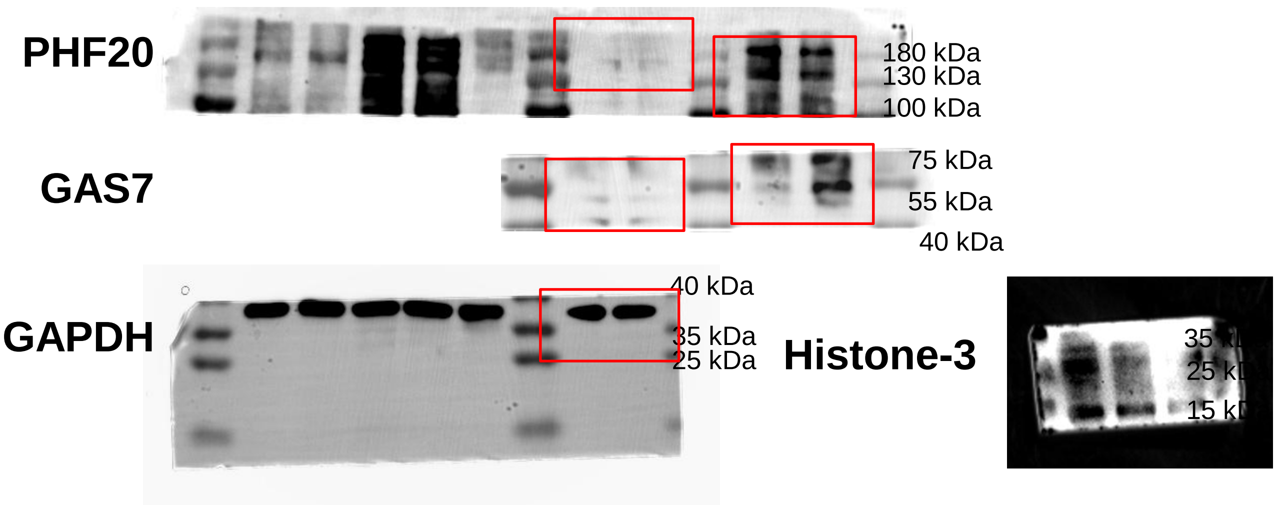

SCL-1

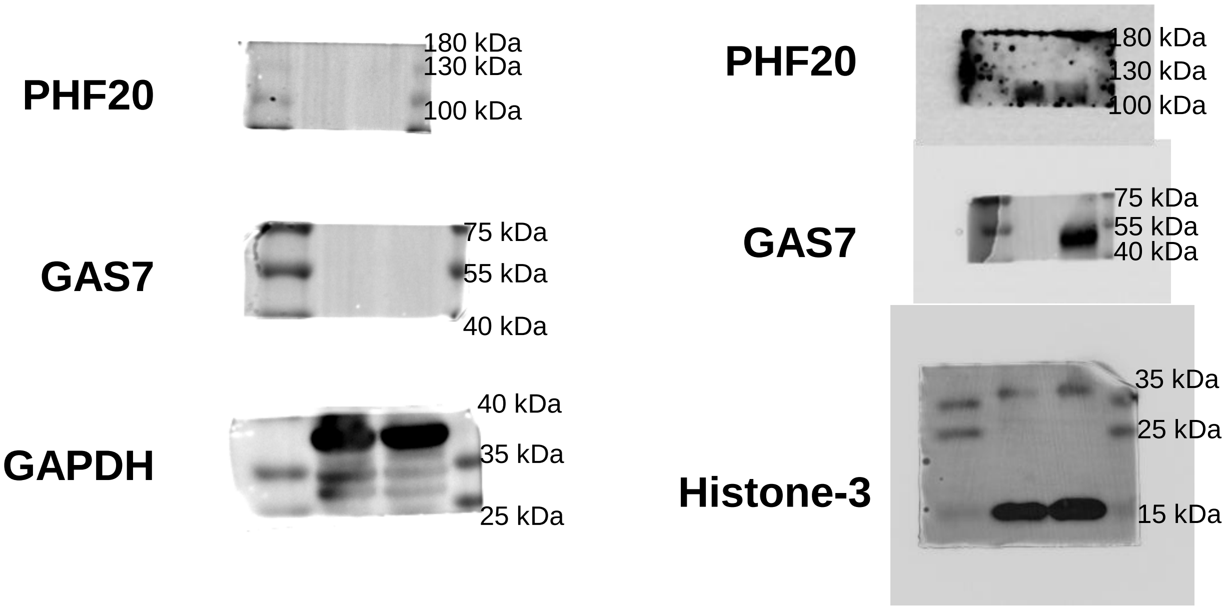

SourceData Figure S4F

A431

GAS7

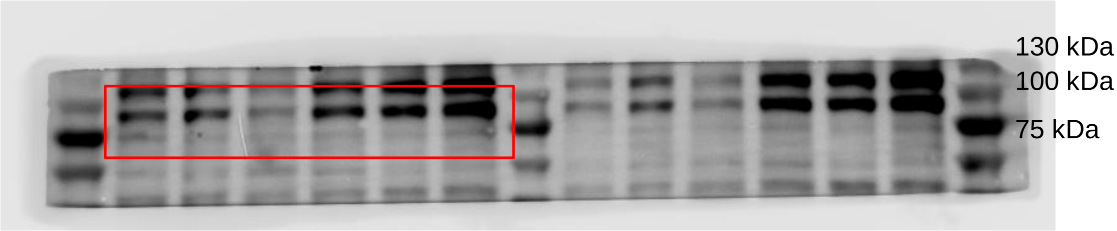

GAPDH

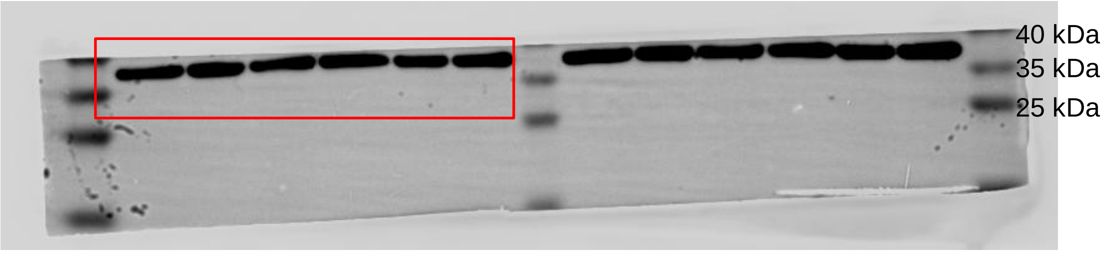

SCL-1

GAS7

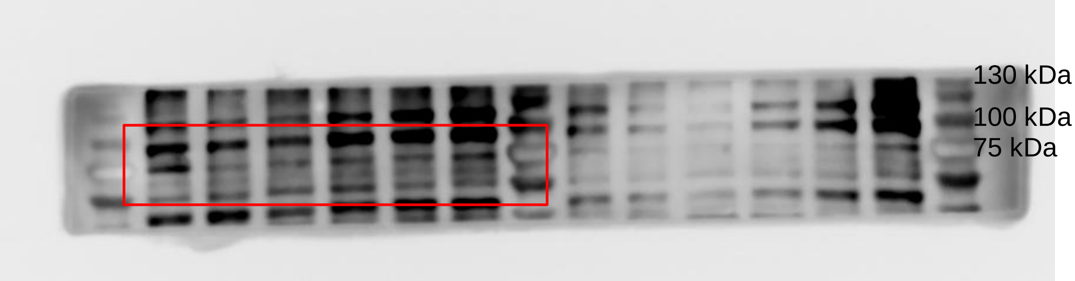

GAPDH

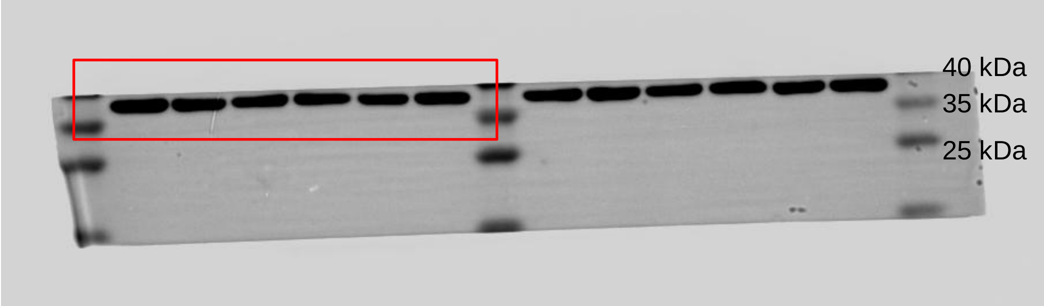

SourceData Figure S5B

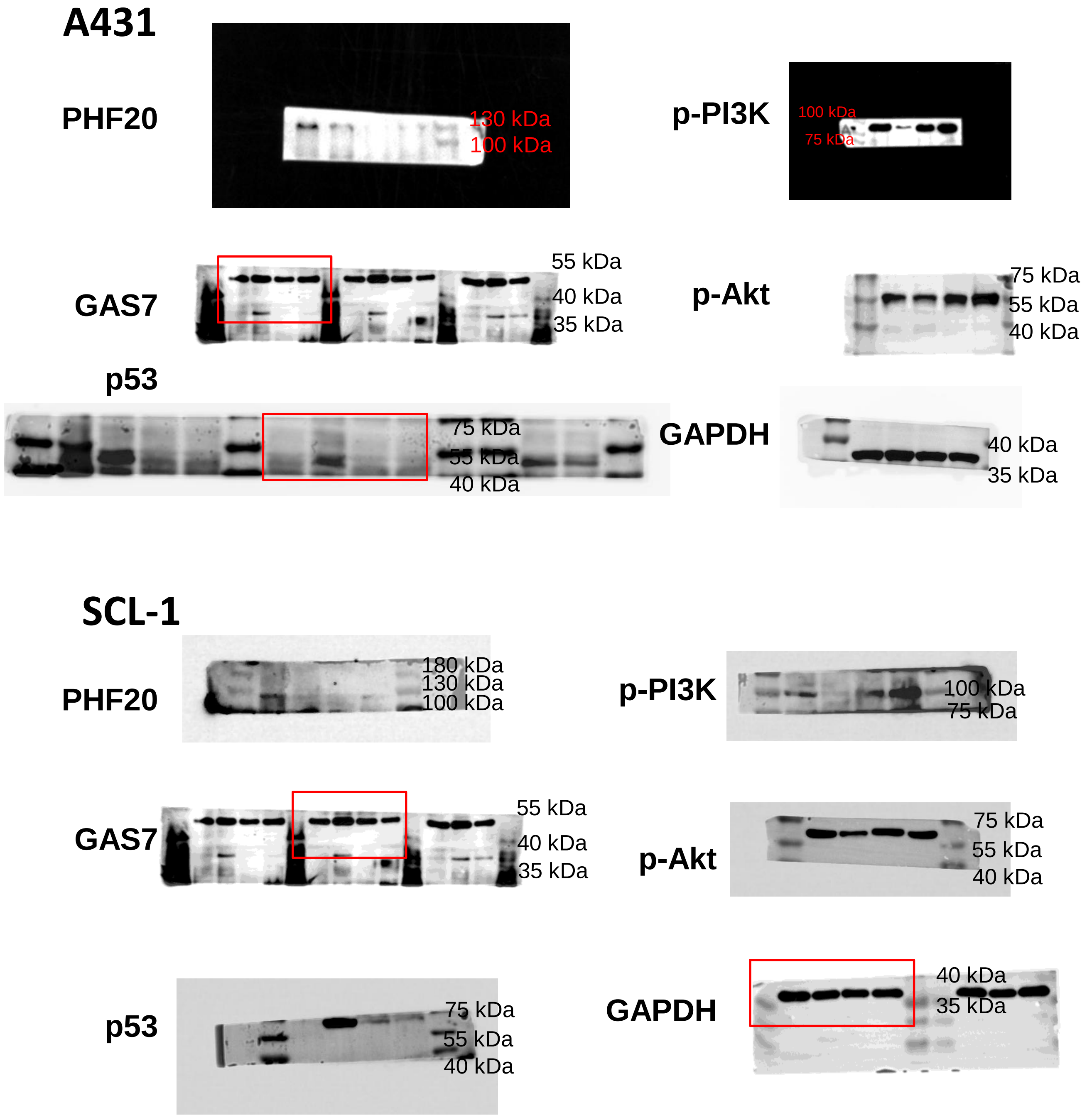

SourceData Figure S6B

A431

Cytoplasm β-Actin

Cytoplasm GAPDH

Nucleus β-Actin

Nucleus LaminB1

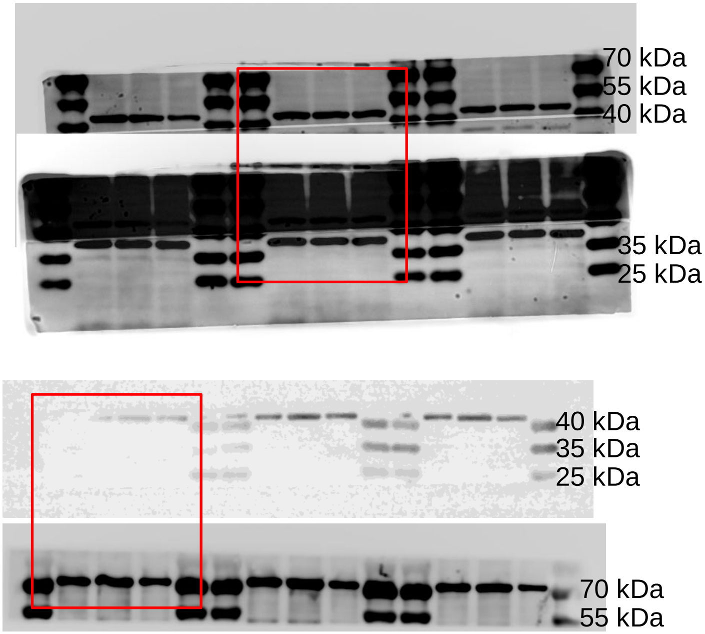

SCL-1

Cytoplasm β-Actin

Cytoplasm GAPDH

Nucleus β-Actin

Nucleus LaminB1

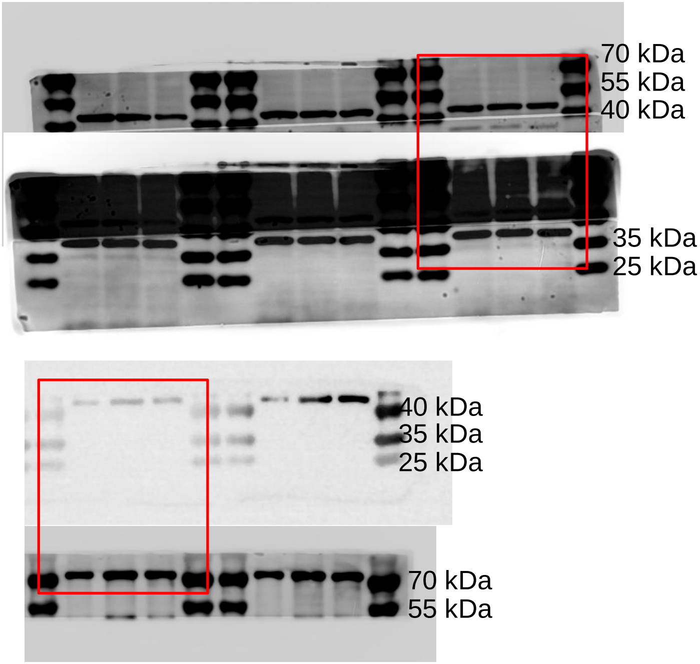

SourceData Figure S7B

A431-Bax

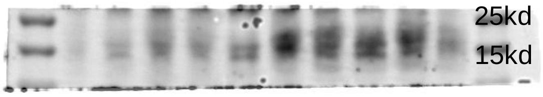

A431-Bcl-2

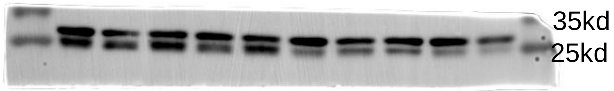

A431-γH2AX

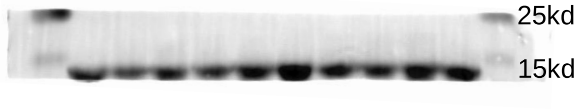

A431-caspase3

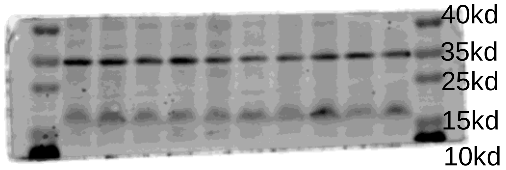

cleaved-caspase3

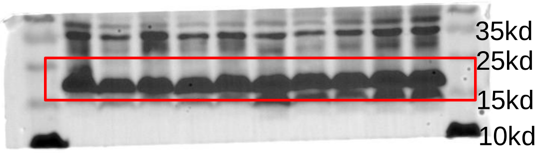

A431-caspase7

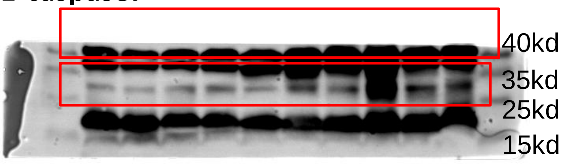

cleaved-caspase7

A431-GADPH

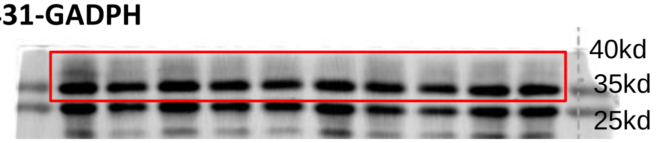

SCL-1-Bax

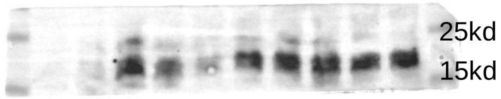

SCL-1-Bcl-2

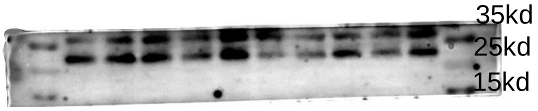

SCL-1-γH2AX

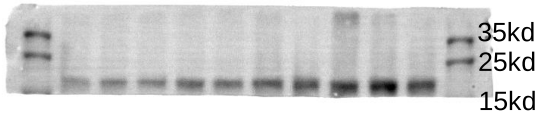

SCL-1-caspase3

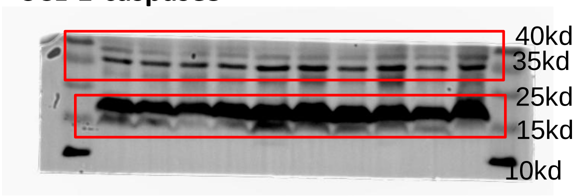

cleaved-caspase3

SCL-1-caspase7

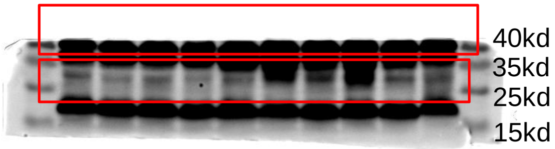

cleaved-caspase7

SCL-1-GADPH

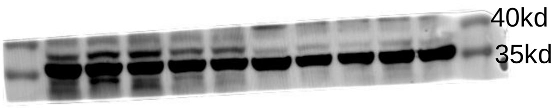

Supplement: Supplementary file 3 — Original western blots [file 41419_2026_8932_MOESM3_ESM.pdf]
